# Angiogenesis-related non-coding RNAs and gastrointestinal cancer

**DOI:** 10.1016/j.omto.2021.04.002

**Published:** 2021-05-15

**Authors:** Zahra Sadat Razavi, Kasra Asgarpour, Maryam Mahjoubin-Tehran, Susan Rasouli, Haroon Khan, Mohammad Karim Shahrzad, Michael R. Hamblin, Hamed Mirzaei

**Affiliations:** 1 School of Medicine, Kashan University of Medical Sciences, Kashan, Iran; 2 Department of Medicine, University of Western Ontario, London, ON, Canada; 3 Department of Medical Biotechnology, School of Medicine, Mashhad University of Medical Sciences, Mashhad, Iran; 4 Department of Pharmacy, Abdul Wali Khan University Mardan, Mardan, Pakistan; 5 Department of Internal Medicine and Endocrinology, Shohadae Tajrish Hospital, Shahid Beheshti University of Medical Sciences, Tehran, Iran; 6 Laser Research Centre, Faculty of Health Science, University of Johannesburg, Doornfontein 2028, South Africa; 7 Research Center for Biochemistry and Nutrition in Metabolic Diseases, Institute for Basic Sciences, Kashan University of Medical Sciences, Kashan, Iran

**Keywords:** gastrointestinal cancers, angiogenesis, circular RNAs, microRNAs, non-coding RNAs, long non-coding RNAs

## Abstract

Gastrointestinal (GI) cancers are among the main reasons for cancer death globally. The deadliest types of GI cancer include colon, stomach, and liver cancers. Multiple lines of evidence have shown that angiogenesis has a key role in the growth and metastasis of all GI tumors. Abnormal angiogenesis also has a critical role in many non-malignant diseases. Therefore, angiogenesis is considered to be an important target for improved cancer treatment. Despite much research, the mechanisms governing angiogenesis are not completely understood. Recently, it has been shown that angiogenesis-related non-coding RNAs (ncRNAs) could affect the development of angiogenesis in cancer cells and tumors. The broad family of ncRNAs, which include long non-coding RNAs, microRNAs, and circular RNAs, are related to the development, promotion, and metastasis of GI cancers, especially in angiogenesis. This review discusses the role of ncRNAs in mediating angiogenesis in various types of GI cancers and looks forward to the introduction of mimetics and antagonists as possible therapeutic agents.

## Graphical abstract

**Figure:**
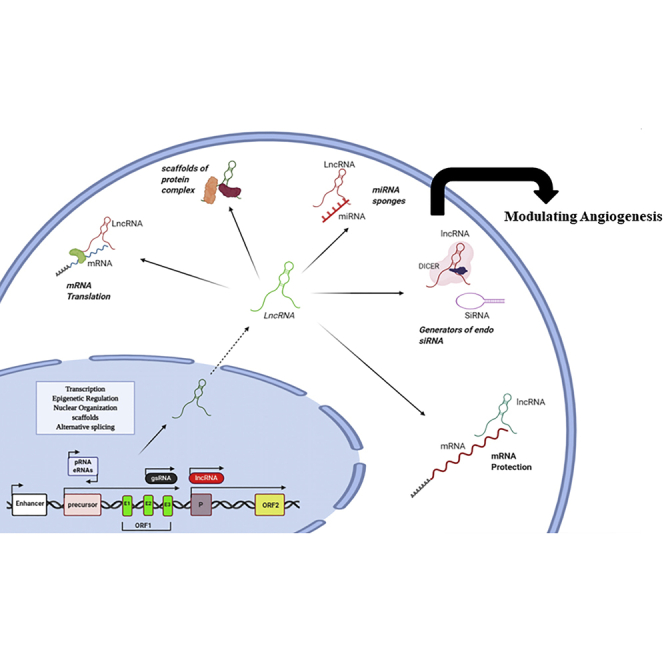

## Teaser

Deregulation of many types of ncRNAs can affect angiogenesis and metastasis in GI cancers. The rapid development of different types of mimetics or antagonists of specific ncRNAs, as well as more effective delivery technologies has raised the possibility to employ ncRNAs as targets to treat metastatic GI cancers.

## Introduction

Gastrointestinal (GI) cancers are among the key reasons for cancer mortality worldwide. Colon, liver, and stomach cancer are the most common types of GI cancer, causing the most deaths.^1^ Although the worldwide cancer statistics show that pancreatic ductal adenocarcinoma (PDAC) and esophageal cancer are less common than other GI cancers, the incidence of pancreatic and esophageal cancer as compared to liver and stomach cancer depends on the region.^1^^,^^2^ Oncogenic mutations are likely to occur in GI tissues (intestines, stomach, and liver) because the epithelial cells are rapidly turned over.^3^^,^^4^ Unfortunately, some GI cancers do not show any symptoms in the early stages. This means that diagnosis often occurs at late stages, which reduces the effectiveness of therapy. Therefore, there is a need to generate newer and more efficient therapeutic approaches to increase patients’ survival. In recent years, many attempts to identify the main mutations in GI cancer cells have been carried out, with the aim to design more effective drugs.^5^^,^^6^ Despite some successes in newer therapeutic approaches, GI cancers remain life-threatening diseases.^7^^,^^8^ Considering its fundamental role in cancer growth and metastasis, angiogenesis has become an attractive target in cancer therapy.

## Angiogenesis and GI cancers

**Figure 1:**
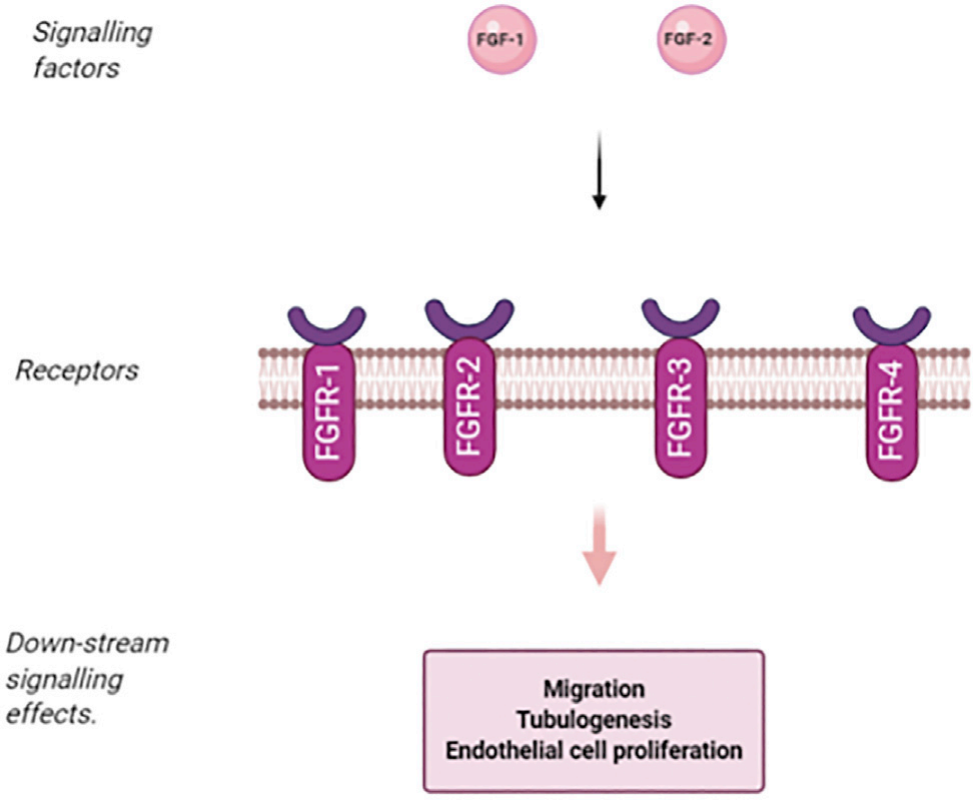
Overview of FGFs and their receptors and related signaling pathways

The expression of VEGF-A is upregulated in colorectal cancer and is associated with colorectal cancer metastasis^9^^,^^10^ and shorter patient survival.^9^^,^^11^^,^^12^ VEGF-A, the first VEGF member to be characterized, was the basis for the development of anti-angiogenesis as a therapeutic strategy, including the clinical development of bevacizumab, a humanized monoclonal antibody targeting VEGF-A. In a pivotal clinical trial, the use of bevacizumab in combination with irinotecan, 5-fluorouracil, and leucovorin was shown to improve the survival of patients with metastatic colorectal cancer (mCRC), resulting in its approval as the first antiangiogenic therapy.^13^ VEGF-A expression is associated with hematogenous and lymphatic spread,^14^^,^^15^ greater microvessel density (MVD),^16^ and a poor prognosis^17^ in gastric cancer (GC). Higher VEGF-A expression in pancreatic cancer is correlated with cancer progression, higher metastatic risk, and poor prognosis.^18^, ^19^, ^20^, ^21^, ^22^ Fibroblast growth factor (FGF) is a family composed of 20 different molecules, which have various biological functions, including the stimulation of angiogenesis.^23^ FGF-1 and FGF-2 are the most studied with regard to angiogenesis.^24^ The functioning of FGFs is mediated through tyrosine kinase receptors (FGFR1–FGFR4)^25^ (Figure 1). Endothelial cells express FGFR-1; however, small amounts of FGFR-2 have also been reported in endothelial cells.^26^ Activation of FGFR can induce the migration, growth, and tube formation of endothelial cells.^27^, ^28^, ^29^, ^30^ Many cancer cell lines secrete FGF-1 and FGF-2. It has been found that the concentration of FGF-2 is increased in the urine of patients with various cancers.^31^^,^^32^ FGFs were upregulated in blood samples of patients with colorectal cancer.^33^ FGFs can increase invasion and proliferation in colon cancer cells.^34^ In colorectal cancer patients, higher FGF-2 levels were associated with an elevated risk of metastasis.^35^ It was reported that there was a positive correlation between levels of FGF expression and stage D of colorectal cancer.^36^ It was also reported that FGFs play a role in cancer cell resistance to chemotherapy.^37^ In addition, serum FGF-2 levels had a predictive value for progression of disease in untreated metastatic colorectal cancer.^38^ FGF was upregulated in GC surgical samples.^39^ It was found that there was a positive association between high expression of FGF and invasion of GC cells^40^ and lymph-node metastasis.^41^ The expression of FGF-2 in GC could predict recurrence after resection.^42^^,^^43^ FGFs have a role in angiogenesis in pancreatic cancer,^44^, ^45^, ^46^ and it was found that the level of FGF could be used for prediction of patient survival and risk of metastasis.^47^ Despite much research, the mechanisms governing dysfunctional angiogenesis are not completely understood. Recently, a number of angiogenesis-related non-coding RNAs (ncRNAs) have been investigated, and they have been found to affect the development of angiogenesis in cancer cells and tumors.^48^

## Angiogenesis-related ncRNAs and GI cancers

ncRNAs are generated via the transcription of various sections of the genome. MicroRNAs (miRNAs or miRs) are an important group of ncRNAs, estimated to be able to regulate 40%–90% of the human genome.^49^ miRNAs and long non-coding RNAs (lncRNAs) can modulate gene expression at the transcriptional as well as post-transcriptional levels. They can also act as epigenetic regulators. It has been found that ncRNAs can inhibit the translation of mRNAs. Moreover, ncRNAs can regulate many biological pathways and subsequently alter the cell fate by stimulating or inhibiting the expression of specific genes.^50^^,^^51^ Recently, many basic research studies have attempted to identify the mechanisms of disease pathogenesis using both living organisms and also *in vitro* and *in silico* systems. These investigations have demonstrated that ncRNAs can play critical functions, for instance in pancreatic cancer development. It has been revealed that miRNAs can affect cancer cell proliferation, migration, invasion, and metastasis.^49^^,^^52^

## Angiogenesis-related microRNAs in GI cancer

### miRNA biogenesis

**Figure 2:**
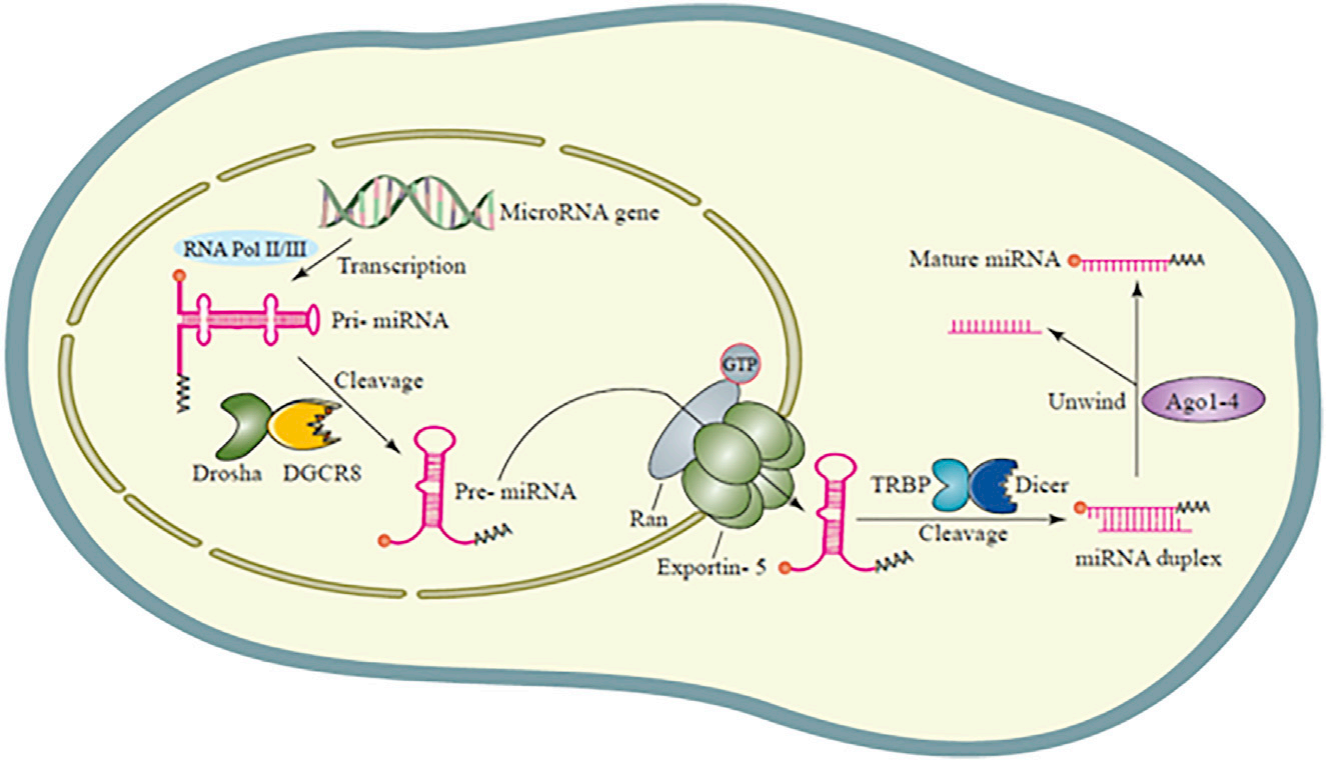
MicroRNA biogenesis

miRNAs are single-stranded non-coding RNAs, which contain 20–22 nucleotides.^53^ miRNAs are transcribed from their specific genes by RNA polymerase II and III, to generate pri-miRNAs (primary miRNAs), which are then cleaved by the Drosha enzyme to form pre-miRNAs (precursor miRNAs).^54^^,^^55^ Pre-miRNAs have a hairpin-like structure, which is cleaved when transported from inside the nucleus into the cytoplasm as a miRNA duplex by the Dicer enzyme to form the mature miRNA structure, which is the active form of miRNA.^56^^,^^57^ The less-stable strand of the miRNA duplex is normally incorporated into the RISC (miRNA-induced silencing complex) in order to regulate protein expression (Figure 2). This regulation is often accomplished by hybridization of the miRNA to the 3′ untranslated region (UTR) of the sequence of its target mRNA.^58^^,^^59^

### Angiogenesis-related microRNAs in colon cancer

miR-145 works in concert with the tumor suppressor p53 and is post-transcriptionally activated by upregulated p53.^60^ miR-145 was first found to be downregulated in colorectal cancer, and then the deregulation of this miRNA was found in lung, breast, ovarian, bladder, nasopharyngeal, prostate, and GC.^61^, ^62^, ^63^ Moreover, miR-145 has a role in smooth muscle cell flexibility and development.^64^ Xu et al.^65^ investigated the effects of miR-145 and its targets, p70S6K1, VEGF, and HIF-1, on angiogenesis in colon cancer. They display that miR-145 lowered in ovarian and colon cancer. The expression of p70S6K1 (ribosomal protein S6 kinase beta-1) is post-transcriptionally suppressed by miR-145. HIF-1 and VEGF, which are the downstream mediators of p70S6K1, were decreased by upregulation of miR-145. Exogenous P70S6K1 rescues the miR-145 suppression of VEGF and HIF-1 levels and restores tumor angiogenesis and tumorigenesis. There is therefore an inverse association between the level of miR-145 and p70S6K1 in colon cancer.^65^

miRNA-27b is located on chromosome 9^66^ and has a role in angiogenesis by increasing endothelial sprouting.^67^^,^^68^ miR-27b plays a role as a tumor repressor by targeting PPARγ in neuroblastoma, although the function of miR-27b, as well as its target in colon cancer, is not completely clear. In one study, Ye et al.^69^ assessed the association between miR-27b and VEGF-C in angiogenesis in colorectal cancer. The expression of miR-27b was downregulated in CRC, and the high expression of miR-27b inhibited tumor growth, cell proliferation, and colony formation. They reported that miR-27b could act as a tumor repressor and inhibit angiogenesis by targeting VEGF-C in colorectal cancer. They also found that DNA hyper-methylation of the CpG islands in miR-27b reduced its expression.^69^

miR-590-5p is one of the newly identified microRNAs, whose functions are not completely understood. However, some studies have shown that this miRNA was downregulated in colorectal cancer. It could also act as an oncogene in cervical cancer but as a tumor suppressor in renal cell cancer.^70^^,^^71^ It is thought that the direct target of miR-590-5p is the mRNA of the transcription factor family NF90, which is transcribed from the ILF3 gene.^72^ The specific protein isoforms are NF90 and NF110. NF90 is about 90 kDa and is also known as NFAR1 or DRBP76. NF110 is about 110 kDa and is also known as NFAR2, TCP110, or ILF3.^72^ NF90 and NF110 are different at the C terminus but homologous in the central region and the N terminus.^72^ NF90 was purified as a DNA binding complex that regulated the IL2 promoter.^73^ NF90 has many functions, such as in protein translation from mRNAs, controlling mitosis, RNA processing, and host resistance to infection.^74^ It was also implicated in the occurrence of angiogenesis of breast cancer. Moreover, the NF90/NF45 complex regulated E6 expression in cervical cancer.^75^ Zhou et al.^76^ studied the effects of miR-590-5p, NF90, and VEGF on angiogenesis in colon cancer. The deletion of miR-590-5p *in vivo* enhanced colorectal cancer progression, while its high expression suppressed tumor growth, angiogenesis, and lung metastasis. NF90 participates in regulating VEGF protein synthesis and is a target of miR-590-5p. High expression of NF90 restored angiogenesis and VEGFA expression. NF90-shRNA reduced tumor development, and the deletion of NF90 decreased the pri-miR-590 and increased miR 590 5p.^76^

miR-19a and miR-19b are about 96% identical to each other and only differ by one nucleotide at position 11. They have a role in some cellular processes and tumor progression.^77^^,^^78^ Chen et al.^79^ investigated the effects of miR-19a and its target, KRAS, on angiogenesis in colon cancer. They generated a KRAS 3′ UTR-Mut by removing the predicted binding site for miR-19a within KRAS. miR-19a inhibited the expression of the gene containing the wild-type KRAS 3′ UTR in HCT116 cells, but the cells with mutant KRAS 3′ UTR were not affected by miR-19a. The high expression of miR-19a decreased the expression of KRAS. In a vascular tube formation assay, the high expression of miR-19a produced anti-angiogenesis effects, which could be rescued via expression of KRAS. They also used a nude mouse xenograft model to study the *in vivo* function of miR-19a in solid tumors. Findings showed that the density of blood vessels and the size of the xenograft tumors grown from HCT116 cells that overexpressed miR-19a were lower in comparison with the controls. Moreover, the levels of VEGF-A and KRAS were decreased.^79^

CXCR4 is a constituent of the GPCR family that is expressed in some epithelial cancers, where it increases proliferation, migration, and angiogenesis.^80^, ^81^, ^82^ Deletion of CXCR4 suppressed CXCL12-induced angiogenesis via downregulating PI3K-AKT, MAPK-ERK, and Wnt-β-catenin signaling pathways.^83^ In a recent study, Fang et al.^84^ assessed the effects of miR-622, CXCR4, and VEGFA on angiogenesis in colorectal cancer. *In vitro* studies showed that the high expression of miR-622 suppressed angiogenesis in CRC and the proliferation, migration, tube formation, and invasion of human umbilical vein endothelial cells (HUVECs). Moreover, the higher expression of miR-622 inhibited angiogenesis in CRC tumors *in vivo*, as detected via the quantification of VEGF-A and Ki67 levels and microvessel density. CXCR4 is a target of miR-622, and higher expression of CXCR4 reversed the VEGFA suppression by miR-622 and also restored the angiogenesis that had been inhibited by miR-622.^84^

Table 1 lists some different angiogenesis-related miRNAs that have been shown to participate in colorectal cancer.

**Table 1:** Angiogenesis-related miRNAs in colon cancer

microRNA	Expression change in CRC	Target	Inhibit or induce angiogenesis	Model (*in vivo*, *in vitro*, human)	Cell line	Reference
miR-1	down	VEGF	inhibit	*in vitro*, human	HT-29, HCT-116, ClonA1, CL-187, SW-620	Zhu et al.^85^
miR-622	up	CXCR4, VEGFA	inhibit	*in vitro*, *in vivo*	Caco-2, HT-29	Fang et al.^84^
miR-19a	up	KRAS	inhibit	*in vitro*	HCT116	Chen et al.^79^
miR-6868-5p	down	FOXM1	inhibit	*in vivo*	HCT8	Wang et al.^86^
				*in vitro*	HCT116	
miR-125a-3p	up	FUT5, FUT6	inhibit	*in vitro*, human	SW480, SW620	Liang et al.^87^
miR-590-5p	down	NF90, VEGFA	inhibit	*in vitro*, *in vivo, in vivo*	HT29, SW620, LOVO, SW480, HCT116	Zhou et al.^76^
miR-17∼92	up	GFBR2, HIF1α, VEGFA	induce	*in vivo*, *in vitro*	HCT116	Ma et al.^88^
miR-145-5p	down	Cx43	inhibit	*in vitro*	SW480	Thuringer et al.^89^
miR-126	down	VEGF	inhibit	*in vitro*, human	LoVo, HT29, SW480, SW620, SW1116, HCT116	Zhang et al.^90^
miR-27b	down	VEGFC	inhibit	*in vitro*, human	SW620, SW480, RKO, HT29, 293T	Ye et al.^69^
miR-885-3p	up	BMPR1A	inhibit	*in vivo*, *in vivo*, human	HT-29	Xiao et al.^91^
miR-150-5p	down	VEGFA	inhibit	human, *in vitro*, *in vivo*	HCT116, SW620, HCT8, HT29, SW480, DLD-1, FHC	Chen et al.^92^
miR-143	down	IGF-IR	inhibit	human, *in vitro*, *in vivo*	SW1116	Qian et al.^93^
miR-107	up	VEGF, HIF-1b	inhibit	human, *in vitro*, *in vivo*	HeLa, HCT116	Yamakuchi et al.^94^
miR-145	down	p70S6K1, VEGF, HIF-1	inhibit	*in vitro*, *in vivo*, human	SW1116, SW480	Xu et al.^65^
miR-181a-5p	down	MMP-14	inhibit	*in vitro*, *in vivo*		Li et al.^95^
miR-503-5p	down	VEGF-A	inhibit	*in vitro*, *in vivo*, human	HT-29, LoVo, HCT116, RKO, SW620	Wei et al.^96^
miR-182-5p	down	VEGF-C	inhibit	*in vitro*, *in vivo*, human	SW620, LoVo, RKO, HT-29, HCT116	Yan et al.^97^
miR-524-5p	up	WNK1	inhibit	*in vitro*, *in vivo*	HT-29, COLO205	Li et al.^98^

### Angiogenesis-related microRNAs in pancreatic cancer

It has been shown that miR-139 can suppress proliferation, migration, and metastasis in some types of cancer.^99^ miR-139 was highly expressed in pancreatic cancer endothelial cells (CECs).

Li et al.^100^ studied the effects of miR-139 and CXCR4 on angiogenesis in pancreatic cancer. Quantitative polymerase chain reaction (qPCR) analysis was applied to quantify the expression pattern of miR-139. The effects of miRNA expression on CEC proliferation, migration, and tube formation were evaluated after transfecting with a specific miRNA suppressor. The expression of fourteen miRNAs was enhanced more than 20 times in the CECs obtained from pancreatic cancer patients. Among these, miR-200c and miR-139 were most overexpressed in CECs. Transfection with inhibitors of miR-200c or miR-139 decreased migration of CECs (all p < 0.05). The average tube length and proliferation were reduced after miR-200c and miR-139 inhibition in three CEC cultures (all p < 0.05).^100^

BCL2L1 and BCL-xL are members of the BCL-2 family^101^ that inhibit apoptosis and autophagy in cancer cells. BCL2L11, also known as Bim, has the opposite function and can stimulate apoptosis by inhibiting BCL2 and BCL-xL. Bim is a pro-apoptotic protein located in the outer mitochondrial membrane, where it promotes the apoptotic cascade.^102^ The role of Bim in pancreatic cancer remains somewhat unclear. Liu et al.^103^ assessed the effects of the miR-24-Bim pathway on angiogenesis in pancreatic cancer. Expression of miR-24 resulted in lower expression of Bim. miR-24 enhanced tumor development and angiogenesis *in vivo* by inhibiting Bim expression.^103^

AGTR1 is expressed in several cancers, such as ovarian carcinoma. Suppression of AGTR1 decreased angiogenesis and cell survival via reducing VEGF expression.^104^ AGTR1 and angiotensin II have functions in endometrial tumor development by inducing VEGF.^105^^,^^106^ Moreover, AGTR1 is upregulated in breast cancer. Inhibition of AGTR1 suppressed proliferation as well as causing G1/S cell cycle arrest.^107^ In breast cancer, AGTR1 enhanced invasion, migration, and metastasis and also stimulated angiogenesis by increasing VEGF-A expression.^108^ Guo et al.^109^ demonstrated the effects of miR-410, AGTR1, and CD31 on angiogenesis in pancreatic cancer. AGTR1 is a target of miR-410, which inhibits its expression, and, conversely, the suppression of miR-410 enhances the AGTR1 expression. Overexpression of miR-410 inhibited cell invasion and growth by reducing AGTR1. In addition, the expression of VEGF and ERK signaling activation were both blocked by miR-410. This was similar to the action of losartan, which acts as an angiotensin II inhibitor. miR-410 blocked the induction of the VEGF and ERK pathways via stimulating angiotensin II. The high expression of miR-410 suppressed angiogenesis *in vivo* by inhibition of CD31. The deletion of the ERK signaling pathway inhibited angiogenesis, cell invasion, and proliferation in pancreatic cancer. Downregulation of miR-410 was detected in pancreatic cancer samples, although there was high expression of AGTR1 in this cancer. Pearson correlation analysis showed an inverse relationship between AGTR1 and miR-410 expression. They concluded that miR-410 inhibited cell migration, growth, invasion, and angiogenesis by reducing AGTR1 expression in pancreatic cancer.^109^

SOCS5 is a member of the SOCS protein family, also known as the SSI protein family.^110^ SOCS5 modulates the function of STAT3 and is mediated by IL-6.^111^^,^^112^ SOCS5 binds to the JAK kinase domain, suppresses the auto-phosphorylation of JAK, and negatively regulates the JAK/STAT3 pathway.^113^

Hu et al.^114^ studied the effects of miR-301a and SOCS5 on angiogenesis in pancreatic cancer. Higher expression of miR-301a in pancreatic cancer was associated with low overall survival. High expression of miR-301a increased angiogenesis, migration, and invasion, while suppression of miR-301a inhibited invasion and decreased orthotopic pancreatic tumor metastasis and growth. SOCS5 has been recognized as a target of miR-301a, because miR-301a inhibited the SOCS5 expression, leading to induction of JAK/STAT3 signaling, and was linked to poor survival of pancreatic cancer patients.^114^

miR-454 acts as an oncogene in several cancers, such as uveal melanoma^115^ and non-small cell lung cancer.^116^ Fan et al.^117^ investigated the effects of miR-454 and LRP6 on angiogenesis in PDAC. Human pancreatic cancer cell lines (PANC-1 and MiaPaCa-2 cells) were transfected with a miR-454-expressing plasmid and assayed for colony formation, cell proliferation, pro-angiogenic markers, cell cycle, and invasion. The effect of miR-454 overexpression on lung metastasis of PDAC was investigated *in vivo*. The high expression of miR-454 suppressed colony formation, cell invasion, and proliferation and arrested the cells at the G2/M stage. miR-454-overexpressing PANC-1 cells contained low levels of VEGF and had a lower ability to induce endothelial cell tube-like formation. LRP6 is a target of miR-454, which can suppress the Wnt/β-catenin pathway activation in PDAC. Ectopic expression of LRP6 reversed the inhibitory effects of miR-454 in PDAC.^117^

Table 2 lists some angiogenesis-related miRNAs involved in pancreatic cancer.

**Table 2:** Angiogenesis-related miRNAs in pancreatic cancer

microRNA	Expression in pancreatic cancer	Target	Effect on angiogenesis (inhibit/induce)	Model (*in vivo*, *in vitro*, human)	Type of cell line	Reference
miR-139	up	CXCR4	inhibit	human		Li et al.^100^
miR200c	up	VEGFA	inhibit	human		Li et al.^100^
miR-24	up	Bim	induce	*in vitro*, *in vivo*	HUVEC	Liu et al.^103^
miR-410	down	AGTR1	inhibit	*in vitro*, *in vivo*, human	PANC-1, MIA-PaCa-2, AsPC-1	Guo et al.^109^
miR-454	up	LRP6	inhibit	*in vitro*, *in vivo*	PANC-1, MIA-PaCa-2	Fan et al.^117^
miR-301a	up	SOCS5	induce	*in vitro*, *in vivo*	PANC-1, BXPC3	Hu et al.^114^

### Angiogenesis-related microRNAs in hepatocellular carcinoma (HCC)

miR29a/b/c are members of the miR-29 family. They are broadly similar to each other, but they have some differences in the target sequence they recognize. They are generally downregulated in HCC.^118^ Some articles have shown that miR-29 has an inhibitory effect on apoptosis, migration, proliferation, and invasion of non-HCC tumor types.^119^, ^120^, ^121^ The miR-29b levels were inversely associated with MMP-2 expression, as well as invasion, metastasis, and angiogenesis. Activation of the MMP-2 enzyme causes degradation of the extracellular matrix (ECM), which then promotes the metastasis and invasion of tumors.^122^ MMP-2 accelerates ECM remodeling and the secretion of ECM-bound growth factors, which can promote the proliferation and migration of ECs. Generally, the overexpression of MMP-2 is observed in HCC.^123^^,^^124^

Fang et al.^125^ investigated the effects of miRNA-29b and MMP-2 on angiogenesis in HCC. They studied the effects of miR-29b on tumor invasion, metastasis, and angiogenesis using Transwell assays and capillary tube formation. Human tumor samples, a Matrigel plug assay, and *in vivo* subcutaneous xenograft tumor growth were used. Loss- and gain-of-function experiments demonstrated that miR-29b inhibited the ability of tumor cells to enhance the formation of endothelial cell capillary tubes and Matrigel invasion. They showed that tumors originating from miR-29b-expressing HCC cells had a lower intrahepatic metastatic capacity and lower microvessel density in mouse models. Experiments showed that MMP-2 was a target of miR-29b. The removal of MMP-2 using a neutralizing antibody or an RNA interference approach duplicated the anti-invasion and anti-angiogenesis properties of miR-29b. They suggested that miR-29b exerted its anti-angiogenic activity by inhibiting the expression of MMP-2 in tumor cells and suppressing VEGFR-2 signaling in endothelial cells.^125^

Bentwich et al.^126^ identified miR-503 for the first time, which was confirmed by Sewer et al.^127^ and cloned by Landgraf et al.^128^ The miR-503 gene is situated on chromosome Xq26.3, and the targets of miR-503 in ECs were found to be cdc25A (cell division cycle 25 homolog) and CCNE1 (G1/S-specific cyclin-E1). miR-503 inhibits endothelial cell function in diabetes mellitus and could promote reparative angiogenesis following limb ischemia.^129^ However, the functions of miR-503 in angiogenesis and cancer development are not completely clear.

In 2013, Zhou et al.^130^ assessed the effects of miR-503, FGF2, and VEGFA on angiogenesis in HCC. miR-503 overexpression lowered angiogenesis *in vitro*, while *in vivo* the expression of miR-503 was decreased by HIF1α. VEGFA and FGF2 are both targets of miR-503 in cancer; therefore, miR-503 plays an anti-angiogenic role in tumorigenesis. The latter suggested a new mechanism for hypoxia-induced VEGFA and FGF2 via HIF1α-induced suppression of miR-503.^130^

Sphingosine kinase 1 (SPHK1) is an enzyme that produces sphingosine-1-phosphate (S1P) and participates in the control of sphingolipid metabolism.^131^ SPHK1 can enhance breast cancer tumorigenesis via increasing S1P and inducing angiogenesis.^132^ The SPHK1/S1P/S1P-receptor axis was confirmed to participate in the angiogenesis associated with liver fibrosis.^133^ Lu et al.^134^ investigated the effects of SPHK1 and miR-506 on angiogenesis in HCC. Database analysis demonstrated that miR-506 could target the 3′ UTR of SPHK1. Using reverse transcriptase-PCR and western blotting, they revealed that miR-506 decreased the protein and mRNA expression of SPHK1. The overexpression of miR-506 in HepG2 cells decreased the S1P content in the supernatant. This supernatant suppressed HUVEC tube formation and decreased angiogenesis. The supernatant from HepG2 cells with high expression of SPHK1 reversed the suppression of angiogenesis. The use of anti-miR-506 increased the S1P production in the supernatant, while knockdown of SPHK1 in HepG2 cells abrogated the anti-miR-506-mediated acceleration of angiogenesis. These studies showed a close relationship between miR-506 and SPHK1 levels in HCC.^134^

miR-146a has a role in several cancers and can regulate the immune system, such as antiviral activity and inflammatory response.^135^, ^136^, ^137^, ^138^, ^139^ The relatively high expression of miR-146a was observed in papillary thyroid carcinoma and could be related to loss of the KIT transcript and Kit protein.^136^ miR-146a could also enhance proliferation in cervical cancer.^140^ Zhu et al.^141^ studied the effects of miR-146a and PDGFRA on angiogenesis in HCC. They examined the miR-146a expression in HUVECs in the presence or absence of HCC cells. PDGFRA is a miR-146a target, and this miRNA could increase angiogenesis by enhancing PDGFRA in HCC. They also found that miR-146a increased expression of PDGFRA in HUVECs via affecting BRCA1. High expression of PDGFRA in HCC clinical samples was correlated with microvascular invasion and predicted a poor prognosis.^141^

miR-126 has a role in cancers by modulating migration, invasion, and proliferation.^142^, ^143^, ^144^ miR-126 is known to be a tumor suppressor, preventing proliferation and enhancing apoptosis in HCC cells.^145^ Gong et al.^146^ investigated the effects of EGFL7 and miR-126 on angiogenesis in HCC. Western blotting and qRT-PCR were used to determine the levels of EGFL7, miR-126, Fas/FasL, ERK, caspase mRNA, and Bcl-2 expression. TUNEL and Cell Counting Kit 8 assays were used to measure apoptosis and proliferation. Flow cytometry was applied to examine cell cycle distribution. A rat model of HCC was established, and the quantity of new blood vessels and tumor weight were measured at 3 weeks post-tumor transplantation. miR-126 was downregulated in HCC, whereas the levels of ERK mRNA and protein and EGFL7 were increased. The high expression of miR-126 inhibited P-ERK, ERK, Bcl-2, and EGFL7 and also enhanced the apoptosis-related proteins caspase-3 and Fas/FasL and suppressed proliferation. Overexpression of miR-126 in nude mice produced fewer blood vessels and reduced the tumor weight. Suppression of miR-126 reduced apoptosis and increased angiogenesis and proliferation.^146^

Table 3 lists some different angiogenesis-associated miRNAs in HCC.

**Table 3:** Angiogenesis-associated miRNAs in hepatocellular carcinoma

microRNA	Expression in HCC	Target	Effect on angiogenesis (inhibit/induce)	Model (*in vivo*, *in vitro*, human)	Type of cell line	Reference
miR-1301	down	BCL9	inhibit	*in vitro*, *in vivo*, human	Hep3B, HepG2, SMMC-7721, Huh-7	Yang et al.^147^
miR-26b-5p	down	VE-cadherin, snail, MMP2	inhibit	*in vitro*, *in vivo*	Bel7402, SMMC7721, HepG2, PLC, LO2	Wang et al.^148^
miR-199a-3p	down	VEGFA, VEGFR2, VEGFR1, HGF, MMP2	inhibit	*in vitro*, *in vivo*	HepG2, SNU449	Ghosh et al.^149^
miR-203a	up	HOXD3, VEGFR	inhibit	*in vitro*, *in vivo*	SMMC-7721, Hep3B	Ghosh et al.^149^
miR-144-3p	down	SGK3	inhibit	*in vitro*, *in vivo*, human	QGY-7703, SK-hep1	Wu et al.^150^
miR-497	down	VEGFA, AEG-1	inhibit	*in vitro*, human, *in vivo*	PLC/PRF/5, SMMC-7721, HepG2, Huh7, SK-HEP-1, Hep3B	Yan et al.^151^
miR-142	down	TGF-β	inhibit	*in vitro*, human	HepG-2, SMMC-7721	Yu et al.^152^
miR-126	up	EGFL7	inhibit	*in vitro*, *in vivo*, human	MMC-7721, MHCC-97H, HCCLM3	Yu et al.^152^
miR-338-3p	down	MACC1, VEGF, β-catenin	inhibit	*in vitro*, human	Hep3B, Huh7, HepG2, Bel-7402, HEK293T	Zhang et al.^153^
miR-638	down	VEGF	induce	*in vitro*, *in vivo*, human	Hep3B, SMMC-7721, HepG2, MHCC-97L, MHCC-97H	Cheng et al.^154^
miR-126	down	EGFL7	inhibit	*in vitro*, *in vivo*, human	HepG2, Bet-7402, SMMC-7721	Gong et al.^146^
miR-210	up	FGFRL1	induce	*in vitro*, *in vivo*, human	HL-7702, SMMC-7721	Yang et al.^155^
miR-182	up	RASA1	induce	*in vitro*, human	SK-HEP-1, HCC-LM3	Du et al.^156^
miR-126	down	Spred1	inhibit	*in vitro*, human		Ji et al.^157^
miR-26a	down	HGF	inhibit	*in vitro*, *in vivo*, human	HUVECs	Yang et al.^158^
miR-126-3p	down	LRP6, PIK3R2	inhibit	*in vitro*, human	HepG2, SMMC-7721, BEL-7402	Du et al.^159^
miR-195	down	VEGF, VAV2, CDC42	inhibit	*in vitro*, *in vivo*, human	MHCC-97L, Huh-7, QGY-7703, MHCC-97H, SMMC-7721	Wang et al.^160^
miR-302a/b/c	down	MACC1	inhibit	*in vitro*	cell line missing	Cao et al.^161^
miR-26a	down	VEGFA	inhibit	*in vitro*, *in vivo*, human	HepG2	Chai et al.^162^
miR-146a	up	PDGFRA	induce	*in vitro*, *in vivo*	HCCLM3	Zhu et al.^141^
miR-506	down	SPHK1	inhibit	*in vitro*, human	HepG2	Lu et al.^134^
miR-98 and miR-214	down	VEGF, Ang-1, MMP-2	inhibit	*in vitro*	HepG2	Yahya et al.^163^
miR-375	up	PDGFC	inhibit	*in vitro*, human	Hep3B, HepG2, Huh1, Huh7	Li et al.^164^
miR-503	down	FGF2, VEGFA	inhibit	human, *in vitro*, *in vivo*	HepG2, LO2	Zhou et al.^130^
miR-29b	down	MMP-2	inhibit	human, *in vitro*, *in vivo*		Fang et al.^125^
miR-200b	down	ERG	induce	*in vitro*, human	Hep3B	Moh-Moh-Aung et al.^165^

### Angiogenesis-related microRNAs in GC

miR-125a is located in chromosome 19q13, and its expression has been found to be low in several cancers, such as breast^166^, ovarian,^167^ and lung cancer^168^ and medulloblastoma^169^. In GC, lower expression of miR-125a was correlated to indicators of malignancy, such as tumor invasion and size.^170^

Dai et al.^171^ assessed the effects of VEGF-A and miR-125a on angiogenesis in GC and reported that miR-125a could affect VEGF-A expression. Low expression of miR-125a enhanced the release of VEGF-A and also increased Akt phosphorylation in HUVECs, angiogenesis, EC migration, and proliferation. miR-125a expression was reduced in GC and was negatively associated with VEGF-A expression (p < 0.05). miR-125a expression was inversely correlated to the microvessel density *in vivo*.^171^

VEGF-A has an important function in the regulation of angiogenesis.^17^ In addition, levels of VEGF-A are correlated with enhanced tumor aggression and decreased survival in patients.^172^^,^^173^ Xie et al.^174^ investigated the effects of miR-1 and VEGF-A on angiogenesis in GC. They evaluated the expression of miR-1 in GC cell lines and the clinicopathological features in 90 paired GC and normal stomach samples. Proliferation and migration assays were used to detect the effects of miR-1 in GC cells. Protein array and bioinformatic analysis was used to identify the miR-1 target. qPCR, ELISA, EC tube formation, and western blotting were used to assess the regulation mechanisms of miR-1. A reporter assay was also used to confirm the presumed binding site of miR-1 on target genes. miR-1 was downregulated in GC specimens. Patients with lower expression of miR-1 had a poor survival compared with those with higher expression of miR-1 (p = 0.0027). miR-1 overexpression in GC suppressed proliferation, EC tube formation, and migration via inhibiting the expression of EDN1 and VEGF-A. Suppression of miR-1 by an antagonist decreased EDN1 and VEGF-A expression in low-malignant GC or non-malignant GC samples.^174^

The PI3K/mTOR/AKT signaling pathway is dysregulated in various cancers. Elements of the AKT/PI3K/mTOR signaling pathway can be mutated or dysregulated in cancer, causing hyper-activation of the pathway and affecting chemosensitivity, apoptosis, proliferation, and other biological processes.^175^

Wu et al.^176^ assessed the effects of miR-616-3p and the AKT/PI3K/mTOR pathway on angiogenesis in GC. They found that the miR-616-3p was overexpressed in GC and was correlated with a poor prognosis. Also, loss-of-function and gain-of-function studies showed that miR-616-3p increased angiogenesis and triggered the epithelial-mesenchymal transition (EMT) in GC.^176^

The mammalian GI tract secretes members of the trefoil factor family, which are a group of small-molecular-weight polypeptides.^177^ TFF1 is a member of the trefoil peptide family, which suppresses GI tumorigenesis. The expression of TFF1 is high in the normal human stomach, where it acts to preserve gastric epithelial function and structure.^178^

Shi et al.^179^ investigated the effects of miR-632 and TFF1 on angiogenesis in GC using serum samples and GC tissues to measure the expression of miR-632 with real-time PCR. A dual-luciferase reporter assay was performed to examine how miR-632 controlled the TFF1 expression. Endothelial cell recruitment and tube formation assays also were used with or without miR-632 treatment. Moreover, *in situ* hybridization assays and western blotting were used to determine markers of endothelial migration and angiogenesis. miR-632 expression was high in GC and inversely correlated with its target TFF1. miR-632 stimulated EC recruitment and tube formation, while recombinant TFF1 reversed the miR-632-induced angiogenesis.^179^

RUNX1, RUNX2, and RUNX3 are members of the Runt family of transcription factors, with key roles in both normal tissue and cancers.^180^ RUNX3 has a role in T cell differentiation, neurogenesis within the dorsal root ganglia, and GC tumorigenesis.^180^

Lee et al.^181^ studied the effects of miR-495, miR-130a, and RUNX3 on angiogenesis in GC and employed bioinformatic and microarray analysis to measure the miR-130a and miR-495 expression. miR-495 and miR-130a were both highly expressed in GC under hypoxic conditions. miR-495 and miR-130a both suppressed the expression of RUNX3 at the protein level but not at the mRNA level. miR-495 and miR-130a suppressed luciferase activity in a reporter assay for RUNX3-3′ UTR binding. miR-130a and miR-495 reduced apoptosis as shown by annexin V-fluorescein isothiocyanate (FITC)/propidium iodide staining as well as flow cytometry. In SNU484 GC cells, the expression of miR-130a and miR-495 was associated with lower levels of RUNX3, p21, and Bim. Antagonistic miRs for miR-495 and miR-130a decreased angiogenesis, as shown by a Matrigel plug assay.^181^

Table 4 lists some angiogenesis-related miRNAs reported to be involved in GC.

**Table 4:** Angiogenesis-related miRNAs in gastric cancer

microRNA	Expression in gastric cancer	Target	Effect on angiogenesis (inhibit/induce)	Model (*in vivo*, *in vitro*, human)	Type of cell line	Reference
miR-632	up	TFF1	induce	*in vitro*	BGC823, MGC803, EAhy926, MKN45	Shi et al.^179^
miR-612	down	FOXM1	inhibit	*in vitro*, *in vivo*	MKN-45, MKN-28, AGS, SGC-7901	Wang et al.^182^
miR-616-3p	up	PTEN/AKT/mTOR pathway	induce	*in vitro*	KN-28, MGC-80, GES-1, HEK293 T, AGS, SGC-7901	Wu et al.^176^
miR-532-5p	down	LINC01410	inhibit	*in vitro*, *in vivo*	MNK-45, SGC-7901, AGS, HGC-27, BGC-23, GES-1	Zhang et al.^183^
miR-26a/b	down	HGF VEGF	inhibit	*in vitro*, *in vivo*	MKN-28, GES-1, AGS, HEK293 T, SGC-7901, MGC-80	Si et al.^184^
miR-1	down	EDN1 VEGF-A	inhibit	*in vitro*, *in vivo*	SGC7901, MKN28, NCI-N87, BGC823, AGS, HGC27	Xie et al.^185^
miR-1228	down	CK2A2	inhibit	*in vitro*, *in vivo*	AGS, SGC-7901, HEK293T	Jia et al.^186^
miR-218	down	ROBO1	inhibit	*in vitro*, *in vivo*	BGC-823, HUVEC-2C, HMVEC	Zhang et al.^187^
miR-520b/e	down	EGFR	inhibit	*in vitro*	SGC-7901, MGC-803	Li et al.^188^
miR-101, miR-27b, miR-128	down	VEGF-C	inhibit	*in vitro*, *in vivo*	MKN-45, SGC-7901, BGC-823	Liu et al.^189^
miR-125a	down	VEGF-A	inhibit	*in vitro*, human	GES-1, AGS, SGC7901, BCG-823, HUVEC	Dai et al.^171^
miR-130a, miR-495	up	RUNX3	induce	*in vitro*	SNU5, SNU16, SNU484, MKN45, MKN1	Lee et al.^181^
miR-506	down	ETS1	inhibit	*in vitro*, human	AGS, SGC-7901, Kato-III, MKN45, BGC-823, HGC-27, MGC-803	Li et al.^190^
miR-874	down	3′ UTR of STAT3	inhibit	*in vitro*, human	AGS, BGC823, MKN28, SGC-7901, GES-1	Zhang et al.^191^
miR-126	down	VEGF-A	inhibit	*in vitro*, *in vivo*, human	SGC-7901, MKN-28, MKN-45	Chen et al.^192^
miR-29a/c	down	VEGF	inhibit	*in vitro*, human	HUVE, SGC790, HEK293T	Zhang et al.^193^
miR-135a	down	focal adhesion kinase (FAK)	inhibit	*in vitro*, *in vivo*, human	MGC-803, MKN45, SGC-7901, BGC-823, MKN1, GES-1	Cheng et al.^194^
miRNA-145	down	3′ UTR of Ets1	inhibit	*in vitro*, *in vivo*, human	SGC-7901, GES-1, MKN-45, HUVEC, AGS	Zheng et al.^195^
miR-616-3p	up	PTEN	induce	*in vitro*, human	MGC-803, MKN-28, AGS, SGC-7901	Wu et al.^176^
miR-506	up	ETS1	inhibit	*in vitro*, human	Kato-III, SGC-7901, BGC-823, HGC-27, MKN45, MGC-803, AGS	Li et al.^190^

### Angiogenesis-related microRNAs in other GI cancers

The expression of miR-377 was low in esophageal squamous cell carcinoma (ESCC) cell lines and also in serum and tumor samples from ESCC patients. miR-377 is located in chromosomal region 14q32, which is sometimes obliterated in ESCC.^196^^,^^197^ The function of miR-377 in cancer is not yet completely clear. CD133 is a marker of tumor-initiating cells (TICs) or cancer stem cells,^198^ but the function of CD133 in ESCC is not completely clear,^199^, ^200^, ^201^, ^202^ and it is uncertain whether CD133 is a TIC marker in ESCC. Li et al.^203^ investigated the effects of miR-377, CD133, and VEGF on angiogenesis in ESCC. miR-377 expression in ESCC was generally low, while the tumor tissue and serum levels of miR-377 were associated with patient survival. Upregulation of miR-377 was inversely correlated with pathologic tumor stage, distant metastasis, resistance to radiotherapy and chemotherapy, and the amount of residual tumor. In the laboratory, high expression of miR-377 suppressed angiogenesis, metastasis, and proliferation in ESCC cell lines, while knockdown of miR-377 had the opposite effects. Furthermore, miR-377 targeted both VEGF and CD133 via binding to their 3′ UTRs. Systemic delivery of a formulated miR-377 mimetic inhibited ESCC tumor development in nude mice, suppressed angiogenesis, and inhibited lung metastasis without any toxicity.^203^

miR-143-3p plays a role in many cancers, including HCC, prostate cancer, lung cancer, and colorectal cancer.^204^, ^205^, ^206^, ^207^ Downregulation of miR-143-3p is correlated with poor clinical outcomes. The VEGF family is composed of VEGFA, VEGFB, VEGFC, VEGFD, VEGFE, and PLGF.^208^ PLGF has a key role in several cancers,^209^ although its activity is limited in normal physiological processes. PLGF stimulates Flt-1 and also synergizes with VEGF effects.^209^ Several small-molecule inhibitors of VEGFR2 and VEGF, including cediranib,^210^ sunitinib,^211^ and vandetanib,^212^ have been approved for cancer therapy. Jin et al.^213^ studied the effects of miR-143-3p, ITGA6, and PLGF on angiogenesis in gallbladder carcinoma. They found that miR-143-3p was an inhibitor of tumor angiogenesis and development. ELISA, antibody arrays, and a PLGF rescue analysis showed that PLGF had a key role in the anti-angiogenic effects of miR-143-3p. Dual-luciferase assays and miRNA target-prediction software were used to demonstrate that ITGA6 functioned as a miR-143-3p target. Western blotting and ELISAs showed that PLGF expression was reduced via the ITGA6/AKT/ PI3K pathway.^213^

Table 5 lists some angiogenesis-related miRNAs in other GI cancers (ESCC and gallbladder cancer).

**Table 5:** Angiogenesis-related miRNAs in other GI cancers.

microRNA	Cancer type	Expression in cancer	Target	Effect on angiogenesis (inhibit/induce)	Model (*in vivo*, *in vitro*, human)	Type of cell line	Reference
miR-377	esophageal	down	VEGF CD133	inhibit	*in vivo*, *in vitro*, human	KYSE30, KYSE70, KYSE150, KYSE270, KYSE410	Li et al.^203^
miR-143-3p	gallbladder	down	ITGA6	inhibit	*in vitro*, *in vivo*, human	GBC-SD, SGC996, NOZ, OCUG-1, EHGB-1	Jin et al.^213^

## lncRNA biogenesis

lncRNAs are over 200 nt in length and are transcribed by RNA polymerase II (Pol II). More than 10,000 different lncRNAs have been estimated to exist in humans.^214^^,^^215^ All mammalian lncRNAs have similar functional, structural, and mechanistic properties. They usually can be spliced and have a poly-A tail.^216^ In addition, they can modulate target gene expression at the transcriptional or post-transcriptional levels and thereby affect many biological and cellular processes.^217^, ^218^, ^219^

**Figure 3:**
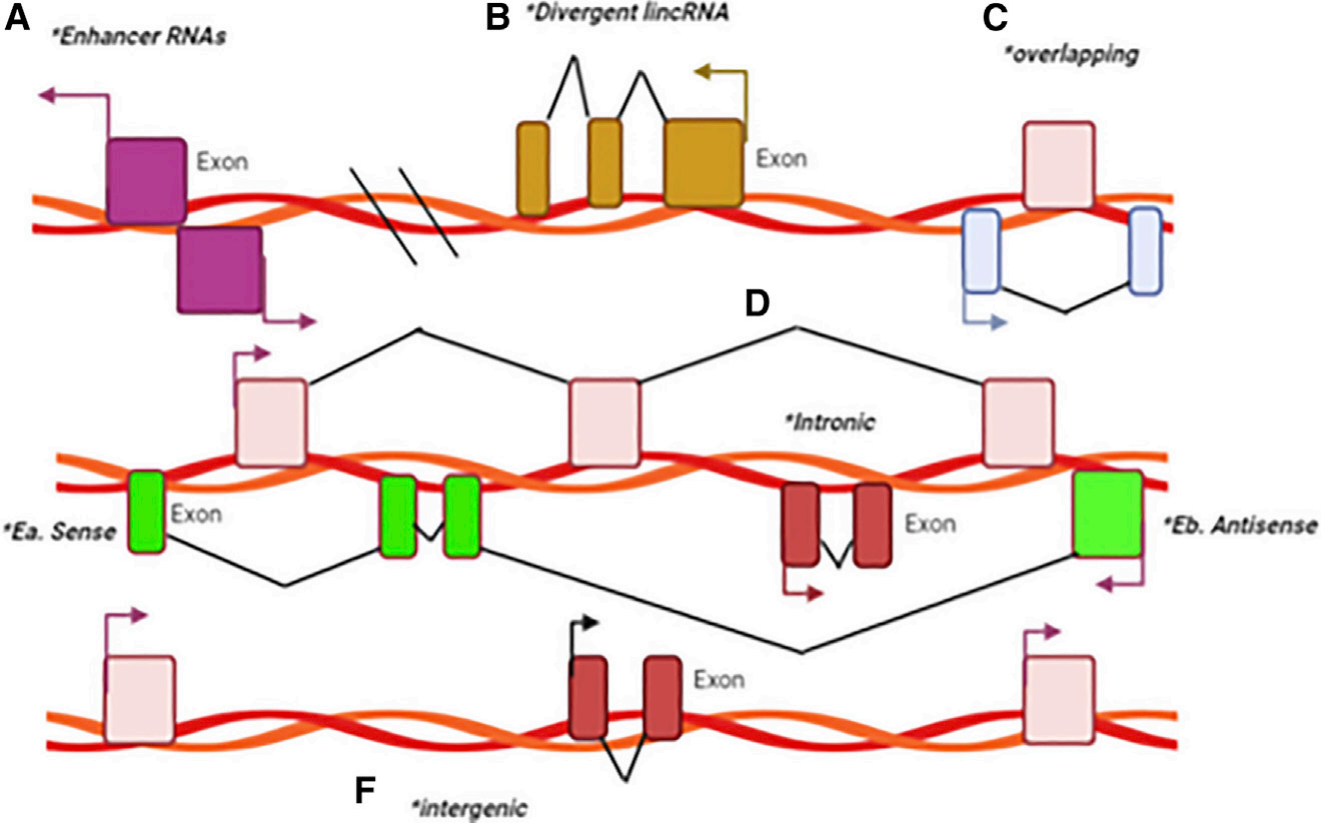
Classification of lncRNAs based on structural origin (A) Enhancer RNAs are transcribed in one (1D-eRNAs) or two senses (2D-eRNAs) by genomic transcriptional enhancers, commonly situated near to protein-coding genes. (B) Divergent lncRNAs: the adjacent protein coding gene and the lncRNA are transcribed on opposite strands. (C) Overlapping lncRNAs: protein-coding genes are overlapped with the lncRNA intron. (D) Intronic lncRNAs: the entire sequence of the lncRNA is contained within the intron of a gene. Ea. sense or Eb. antisense types: the lncRNA is located between the exons of a different transcript on the antisense/sense strand. (F) Intergenic lncRNAs: the sequence of lncRNA is contained within two distinct genes as a single unit.

Spurlock et al.^220^ categorized lncRNAs according to the structural source (Figure 3). In overlapping lncRNAs, the protein-coding gene sequence is overlapped with the lncRNA intron.^220^^,^^221^ In divergent lncRNAs, the adjacent protein coding gene and the lncRNA are transcribed on opposite strands.^222^ In intronic lncRNAs, the entire sequence of the lncRNA is the same as the intron of a gene.^223^ In intergenic lncRNAs, the sequence of the lncRNA is distributed between two distinct genes.^224^ Intergenic lncRNAs can either be sense^225^ or antisense^226^ types, where the lncRNA sequence is located between the exons of a different transcript on the antisense/sense strand.^227^^,^^228^ Finally, enhancer RNAs can be transcribed in one sense (1D-eRNAs) or in two senses (2D-eRNAs) as genomic transcriptional enhancers, commonly situated near to protein-coding genes.^229^

**Figure 4:**
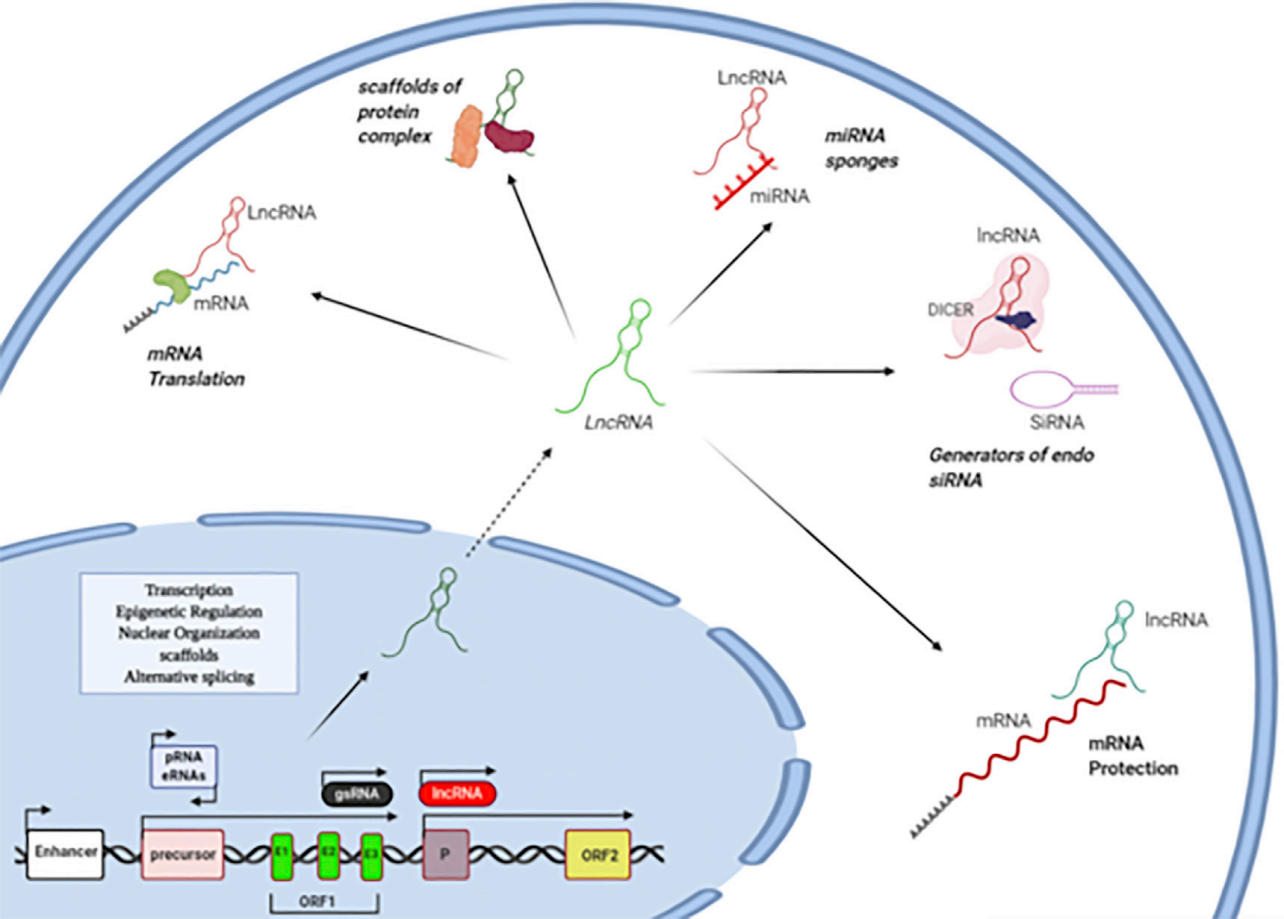
lncRNA classification based on their function lncRNAs are involved in mRNA transcription, epigenetic modulation, nuclear organization, and altered splicing at the nuclear level. In the cytoplasm, lncRNAs can act as enhancers for mRNA translation, miRNA sponges, generators of endogenous siRNA, scaffolds for protein complexes, and protectors of mRNA.

Numerous factors can regulate the function of lncRNAs. For example, RNA-binding proteins (RBPs) have been demonstrated as important regulators of lncRNAs. An important regulatory mechanism of lncRNAs is the post-transcriptional regulation by RBPs. Some cancer-associated lncRNAs have been found to be regulated by the interaction with RBPs, such as human antigen R (HuR), ARE/poly(U)-binding/degradation factor 1 (AUF1), insulin-like growth factor 2 mRNA-binding protein 1 (IGF2BP1), or tristetraprolin (TTP).^230^ Some lncRNAs can function as scaffolds during formation of subcellular structures, such as nuclear bodies. These nuclear bodies are formed by phase separation of RNA-binding proteins with a prion-like domain, low complexity region, or intrinsic disordered region. The scaffold ncRNAs forming these nuclear bodies are also referred to as architectural RNAs (arcRNAs). They can bind to and assemble RNA-binding proteins and thereby induce liquid-liquid phase separation.^231^ Moreover, it has been found that the functions of lncRNAs depend on their subcellular localization.^217^ By using RNA fluorescence *in situ* hybridization in human cell lines, a broad range of subcellular localization patterns, either in the nucleus or the cytoplasm, have been demonstrated.^232^ Nonetheless, it is usual to list lncRNAs based on their similar mechanisms of action^233^ (Figure 4).

Some lncRNAs play a role in the nuclear structure by regulating the structure of nuclear interchromatin granules, paraspeckles, and speckles.^234^ Some other roles of nuclear lncRNAs are the regulation of gene expression via epigenetic activities as well as chromatin-modification factors.^235^ In addition, some kinds of stable lncRNAs, as well as circular RNAs (circRNAs) and competing endogenous RNAs (ceRNAs), are located together within the cell, where they act as sponges or decoys to remove their target miRNAs and thereby alter gene expression.^236^ The ceRNA hypothesis suggests an intrinsic mechanism to regulate biological processes. However, whether the dynamic changes of ceRNAs can affect miRNA activities remains controversial.^237^^,^^238^ The ceRNA hypothesis postulates that RNAs that share miRNA response elements (MREs) in their 3′ UTRs can influence the expression of miRNAs and induce gene silencing. Several recent studies have demonstrated that lncRNAs can contain MREs and interact with other RNA transcripts such as ceRNAs. The complex crosstalk involving ceRNAs has been found in many different cancer types, including GC.^237^^,^^238^ A more updated concept that builds on this previous idea is the target-directed miRNA decay (TDMD), which involves the direct degradation of the miRNA rather than its transient binding to a complementary sequence. This mechanism is triggered by target RNAs with extensive complementarity between the miRNA and the target, especially at the 3′ end, triggering the dissociation of the miRNA from the AGO PAZ domain.^239^, ^240^, ^241^, ^242^, ^243^ Above all, functional interactions and disequilibrium of ceRNA networks (ceRNETs) may contribute to disease pathogenesis.

lncRNAs have a crucial role in transcription by helping the assembly of transcriptional repressors or activators to modulate transcription.^244^ lncRNAs can also regulate gene expression after transcription by modulating RNA-binding proteins, which affect translation and splicing, as well as altering mRNA translation or mRNA stability.^245^^,^^246^ Additionally, some lncRNAs can post-transcriptionally regulate protein expression and affect the fate of proteins by increasing ubiquitination.^247^

### lncRNAs and angiogenesis in GI cancer

The lncRNA SNHG6 is situated on chromosome 8q13.1 and comprises 5 transcripts (SNHG6-201 to SNHG6-205). High expression of SNHG6 has been observed in several different cancers, such as HCC,^248^ CRC,^249^^,^^250^ lung adenocarcinoma,^251^ and breast cancer.^252^ Overexpression of SNHG6 was associated with enhanced tumor progression and poor survival in subjects with cholangiocarcinoma (CCA). SNHG6 has a role in cell apoptosis, invasion, proliferation, and migration *in vitro*, and enhanced tumor growth *in vivo*, but the role of SNHG6 in CCA remains uncertain. Wang et al.^253^ studied the effects of SNHG6 and E2F8 on angiogenesis in CCA. They found that the expression of SNHG6 was generally high in CCA. Moreover, ectopic expression of SNHG6 stimulated cell cycle progression, proliferation, angiogenesis, and migration in CCA, and the deletion of SNHG6 suppressed several cellular processes. Several articles have suggested that SNHG6 could compete with E2F8 to bind to miR-101-3p.^253^ The thrombospondin family consists of 5 members, which play a key role in several cellular processes.^254^ THBS1 (also known as TSP1) is an endogenous anti-angiogenic factor that inhibits tumor development by suppressing angiogenesis.^255^^,^^256^ The high expression of THBS1 on T cells also suppressed angiogenesis and inhibited tumor development.^257^ Wang et al.^258^ assessed the effects of BZRAP1-AS1 and THBS1 on angiogenesis in HCC. Screening of the lncRNA genes that were expressed in HCC suggested a candidate was BZRAP1-AS1. Microarray-based data analysis and qRT-PCR were employed to measure the expression of THBS1 and BZRAP1-AS1 in HCC. Bisulfite sequencing and methylation-specific PCR were used to measure the gene methylation level. Chromatin immunoprecipitation (ChIP) assays and ribosome-inactivating protein RNA pull-down were performed to assess the interactions between BZRAP1-AS1, DNMT3b, and THBS1. The *in vitro* role of DNMT3b, THBS1, and BZRAP1-AS1 in angiogenesis and *in vivo* tumorigenesis were assessed via gain- and loss-of-function tests. BZRAP1-AS1 was found to be overexpressed in HCC. Under-expression of BZRAP1-AS1 in HCC suppressed angiogenesis, proliferation, and migration of HUVECs. Knockdown of BZRAP1-AS1 suppressed angiogenesis and HCC tumor development *in vivo*, by upregulating THBS1.^258^

The lncRNA OR3A4 was recently discovered to be a regulator of GC and breast cancer.^259^^,^^260^ Guo et al.^259^ reported that OR3A4 could enhance invasion, migration, HUVEC tube formation, and angiogenesis in GC. Because the function of OR3A4 in HCC was unclear, Li et al.^261^ studied the effects of OR3A4 and the mTOR/AGGF1/Akt pathway on angiogenesis in HCC. OR3A4 was overexpressed in HCC cell lines and tissues. Loss-of-function assays confirmed OR3A4 as a promoter for HCC development and angiogenesis. They used Kaplan-Meier analysis and qRT-PCR to find a correlation between AGGF1 and OR3A4 expression in tumor samples and poor prognostic outcomes in HCC patients. Spearman’s correlation curve and western blotting demonstrated a positive correlation between OR3A4 and the AGGF1 level. Rescue assays showed that OR3A4 enhanced cancer development and angiogenesis in HCC by affecting AGGF1/Akt/mTOR.^261^

One member of the trypsin-like serine protease family is human kallikrein-related peptidase 4 (KLK4).^262^^,^^263^ The level of KLK4 expression was associated with the development and progression of prostate cancer.^264^ Cui et al.^265^ showed that KLK4 could control the Wnt/β-catenin pathway to enhance oral squamous cell carcinoma. Tang et al.^266^ studied the effects of LINC01314 and KLK4 on angiogenesis in GC. Data analysis and microarrays were used to screen for expression of several lncRNAs in GC, and they detected a binding relationship between KLK4 and LINC01314. The human GC cell line SGC-7901 was modified by overexpression or silencing of LINC01314 or KLK4 to examine how LINC01314 could affect cellular processes in GC. Wnt-1, KLK4 cyclin D1, β-catenin, E-cadherin, and N-cadherin levels were measured, and cell invasion and migration were assessed. Then, microvessel density, tumor weight, and VEGFR-3 and VEGF-C expression in tumors were evaluated. KLK4 was found to be a target gene of LINC01314. Silencing of KLK4 or overexpression of LINC01314 led to less invasion and migration of GC cells and correlated with lower expression of β-catenin, Wnt-1, N-cadherin, and cyclin D1, while E-cadherin was increased. Moreover, the MVD and tumor weight of the transplanted tumors were lower, and angiogenesis was inhibited, accompanied by downregulation of VEGFR-3 and VEGF-C.^266^

Table 6 lists some lncRNAs related to angiogenesis in GI cancers.

**Table 6:** Angiogenesis-related lncRNAs in GI cancers

Cancer	lncRNA	Expression in cancer	Target	Effect on angiogenesis (inhibit/induce)	Model (*in vivo*, *in vitro*, human)	Type of cell line	Reference
Colon cancer	HNF1A-AS1	up	OTX1	induce	*in vivo*, *in vitro*	HCT116, SW620	Wu et al.^267^
	SUMO1P3	up	VEGFA	induce	*in vitro*, *in vivo*, human	HT29, HCT116, SW480, SW620, LoVo	Zhang et al.^268^
Pancreatic cancer	JHDM1D-AS1	up	HGF, FGF1	induce	*in vitro*, *in vivo*	PANC-1	Kondo et al.^269^
Gastric cancer	LINC01410	up	NF-κB	induce	*in vitro*, *in vivo*, human	HGC-27, BGC-23, AGS, MNK-45, SGC-7901	Zhang et al.^183^
	LINC01314	down	KLK4	inhibit	*in vitro*, *in vivo*	SGC-7901	Tang et al.^266^
	PVT1	up	VEGFA	induce	*in vitro*, *in vivo*, human	BGC-823, MNK-45, GES-1, SGC-7901, HGC-27, HUVEC, AGS, SUN-638	Zhao et al.^270^
Hepatocellular carcinoma	LINC00488	up	TLN1	inhibit	*in vitro*, *in vivo*, human	Huh-7, Hep3B, HCCLM3, MHCC97	Gao et al.^271^
	lncRNA-OR3A4	up	AGGF1	induce	*in vitro*, human	Huh7, SMMC-7721, HepG2, Hep3B	Li et al.^261^
	BZRAP1-AS1	up	AS1, BZRAP1	inhibit	*in vivo*, *in vitro*, human	HuH-7, BEL-7405, SK-HEP-1, HCCLM3, LI7, BCLC-9	Wang et al.^258^
	UBE2CP3	up	ERK1/2/HIF-1α/VEGFA	induce	*in vitro*, *in vivo*, human	HepG2, SMMC-7721	Lin et al.^272^
	lncRNA MVIH	up	PGK1	induce	human		Yuan et al.^273^
	MALAT1	up	VEGF-A	induce			Hou et al.^274^
Cholangiocarcinoma	SNHG6	up	E2F8	induce	*in vitro*, *in vivo*, human	HCCC-9810, RBE	Wang et al.^253^

## circRNA biogenesis

circRNAs are non-coding RNAs that can be produced by two possible mechanisms for closed-loop generation. These models were suggested by Jeck et al.:^275^ (1) circularization carried out by intron-pairing, and (2) lariat-driven circularization. Both of these have been broadly accepted to occur. The generation of intron-pairing-driven circularization results from the pairing between complementary bases within the sequence of various introns. This brings adjacent exons to close proximity, and then the spliceosome cuts away the neighboring exons and the introns combine to form the circRNA configuration. The lariat-driven circularization is based on a covalent binding between the donor and splice acceptor. It results in a circRNA that includes the exon lariat.^275^ Another subtype of circRNA is called intron circRNA, which has recently been discovered.^276^ The 7-nucleotide G-rich element and 11-nucleotide C-rich element within the parent gene of the intron circRNA are merged to create a circular structure, which is then spliced by the spliceosome.^276^ The spliceosome plays a key role in circRNA biogenesis, relying on *trans*-acting factors and *cis*-regulatory elements.^277^

In one study by Zhang et al.,^278^ four thiopurines were used to tag newly created RNA to demonstrate that low levels of circRNA could be a byproduct of imperfect pre-RNA splicing. Transcription mediated by RNA polymerase II (pol II) occurred simultaneously with the generation of circRNAs, showing that rapid extension of the strand may improve the reverse splicing of the complementary paired sequences. Moreover, the activity of pol II is rigorously regulated by cis-regulatory elements.^278^ It was suggested that circRNA biogenesis may be post-transcriptional in nature.^279^ In addition to pol II and pre-RNA, circRNA biogenesis is also controlled by a variety of enzymes, proteins, active elements, and intron sequences. Previous research indicated that RNA-binding proteins could also play a role in circRNA generation, as a distinct inhibitory mechanism noted in tumors during the EMT.^280^ The RNA helicase DExH-box helicase 9 (DHX9) is specific for the reverse repeat Alu element, which is required for the modulation of RNA post-transcriptional splicing.^281^ Alu elements are a group of functional sequences commonly observed in primates and are closely involved with circRNA biogenesis.^281^ The levels of DHX9 expression influenced the generation of splicing products, and its deletion was found to promote circRNA biogenesis.^281^ Furthermore, exon circularization is a dynamic process that is controlled by adjacent introns. Indeed, flanking and individual introns can regulate circRNA biogenesis via base-pairing.^282^ Alterations to the mRNA structure have been found to modulate alternative splicing, transcription, advanced structure, translation, and stability. Moreover, Tang et al.^283^ discovered that N6-methyladenosine (m6A) could improve circRNA generation in open reading frames within mouse male germ cells. Moreover, the tissue levels of other circRNAs could also unfavorably affect circRNA biogenesis^284^.

### circRNAs and angiogenesis in GI cancers

Hox genes can regulate cell differentiation and development, receptor signaling, apoptosis, and angiogenesis in cancer.^285^ The function of HOXC6 in PDAC is not clear, but high expression of HOXC6 in prostate cancer was correlated with disease development.^286^ Shi et al.^287^ assessed the effects of hsa_circ_001653 and the human homeobox on angiogenesis in PDAC. Hsa-circ-001653 expression was evaluated in 83 paired tumor and normal tissue samples. Assays for cell cycle, cell viability, apoptosis, and invasion were used to examine phenotypic changes in PDAC cells. HUVEC tube-like formation was evaluated in the presence of PDAC cells. In addition, crosstalk between miR-377 and hsa_circ_001653 HOXC6 was investigated using Ago2 immunoprecipitation, northern blot analysis, and a dual-luciferase reporter assay. Human PDAC cells were inoculated into nude mice for analysis of *in vivo* tumor growth. Hsa_circ_001653 expression was high in PDAC samples. Hsa_circ_001653 knock-down in PDAC cells using RNA interference suppressed cell-cycle progression, cell viability, invasion, and *in vitro* angiogenesis and showed a pro-apoptotic effect. When human PDAC cells were inoculated into nude mice, suppression of hsa_circ_001653 had a therapeutic effect on tumor growth.^287^

HN1 (hematological and neurological expressed 1) protein was first identified in mouse embryonic tissues^288^ and has a role in several diseases.^289^ HN1 was correlated with a poor prognosis in HCC patients.^290^ Pu et al.^291^ investigated the hsa_circ_0000092 and HN1 effects on angiogenesis in HCC. The levels of miR-338-3p, HN1, and hsa_circ_0000092 expression in HCC were examined. RNA pull-down, dual-luciferase reporter, and northern blot assays were used to detect the relationship between miR-338-3p, hsa_circ_0000092, and HN1. A group of mimetics, suppressors, or small interfering RNA (siRNA) plasmids were delivered into HCC cells to confirm the ability of HN1, miR-338-3p, and hsa_circ_0000092 to regulate *in vitro* angiogenesis, cell migration, proliferation, and invasion. The function of hsa_circ_0000092 in HCC tumor development was investigated by silencing hsa_circ_0000092 with siRNA. The expression levels of hsa_circ_0000092 and HN1 in HCC were high, while the expression of miR-338-3p was downregulated. Hsa_circ_0000092 could bind to miR-338-3p in order to increase HN1 expression. Both the downregulation of hsa_circ_0000092 or the upregulation of miR-338-3p were observed to inhibit angiogenesis, cell invasion, proliferation, and migration in HCC cells *in vitro*, by downregulating HN1. Knock-down of hsa_circ_0000092 inhibited HCC tumor development *in vivo*.^291^

Table 7 lists some circRNAs reported to be related to angiogenesis in GI cancers.

**Table 7:** Angiogenesis-related circRNAs in GI cancers

Cancer	CircRNA	Expression in gastric cancer	Target	Effect on angiogenesis (inhibit/induce)	Model (*in vivo*, *in vitro*, human)	Type of cell line	Reference
Pancreatic ductal adenocarcinoma	hsa_circ_001653	up	HOXC6	inhibit	*in vitro*, *in vivo*, human	Capan-2 (ZY-H431), SW1990(ZY-H338), PANC1 (ZY-H147), BxPC3 (ZY-H145)	Shi et al.^287^
Hepatocellular carcinoma	hsa_circ_0000092	up	HN1	induce	*in vitro*, *in vivo*, human	Hep3B, LM3, MHCC97L, SK-hep1, HepG2	Pu et al.^291^
	ircRNA-100338	up	MMP2, MMP9	induce	*in vitro*, *in vivo*, human	Hep3B, MHCC97L, MHCC97H, HLE, Huh7, BEL7402, SMCC7721 HCCLM3 and HCCLM6	Huang et al.^292^
	CircGFRA1	up	miR-149	induce	*in vitro*, human	SK-HEP-1, Huh6, Huh7, HCCLM3	Yu et al.^293^

## Future for therapeutic ncRNAs in GI cancer

ncRNAs are involved in the regulation of almost all physiological processes, such as cell development, differentiation, proliferation, and apoptosis. In this context, ncRNAs could have great potential as new biomarkers for diagnosis and prognosis and in therapeutic approaches for cancers, including GI cancer. Recently, new methods and tools have been developed to detect and quantify cancer-regulated ncRNAs. For example, the use of multiplexed qRT-PCR, microarrays, or next-generation sequencing (NGS)-based genome-wide approaches have helped to provide a complete picture of the expression level of many ncRNAs.^294^ Moreover, bioinformatic approaches and various databases have enabled the discovery of thousands of novel ncRNAs in cancers, including GI cancer. These databases include CRlncRNA, Lnc2Cancer, and LncRNADisease.^295^, ^296^, ^297^

The ncRNAs could be an attractive new type of therapeutics, especially against undruggable targets for the treatment of GI cancer.^298^ Several oncogenic ncRNAs can promote adverse drug reaction resistance in GC. These include miRNAs, such as miR-27a, miR-19a/b, and miR-135a-5p; lncRNAs, such as HOTAIR, CASC9, MRUL, UCA1, D63785, NEAT1, and HULC; and circRNAs, such as circAKT3 and circFN1.^299^ Therefore, ncRNAs could provide a new approach for better clinical decision making. For example, miRNAs can mediate potent and specific gene silencing, making them attractive therapeutic tools. To date, the greatest efforts in this setting have been to explore the potential application of ncRNA-based therapeutics for cancer.^300^

Despite the potential of ncRNAs in cancer therapy, many challenges still remain, including rapid degradation and clearance, poor cellular uptake, off-target effects, and immunogenicity. Rational design, chemical modifications, and improved delivery carriers offer significant opportunities to overcome these challenges.^301^ Moreover, the accurate mechanisms of ncRNAs in particular processes, such as angiogenesis, have not been completely characterized. Despite these limitations, future research could improve ncRNA properties to overcome these challenges. For example, miRNA delivery is being addressed in many different ways, such as with chemical modifications and nanotechnology-based delivery vehicles.^302^

The expression patterns of ncRNAs and mRNAs using *in silico* approaches are always used before wet-lab validation experiments. However, it is worth noting that the gene expression profiles in the human population (particularly in processes such as angiogenesis) are extremely heterogenous, due to different genetic backgrounds, environmental exposure, dietary considerations, and overall health status of the tissue donors. A weak association or a non-significant association does not necessarily mean that the expression of a particular ncRNA and an mRNA are not related.^303^

In addition to *in silico* prediction and correlation analysis of the relationship between the expression of a miRNA and the expression of its target mRNA, *in vitro* experiments employing the transfection of ectopic miRNA mimics or inhibitors into cells is a commonly used approach to validate the function of a particular miRNA. However, in some circumstances, the ectopic miRNA mimics or inhibitors in cells are over-expressed to a level that is often much higher than the endogenous level of the miRNA, suggesting the possibility of an exaggerated effect in the study. On the other hand, the concentration of a miRNA may not reach the level that is necessary within an intracellular compartment to allow its function to suppress target genes (in reality, subcellular concentrations of specific miRNAs are very difficult to measure), which could lead to the effect of the miRNA *in vitro* being underestimated or completely ignored. Furthermore, almost all *in vitro* studies have demonstrated the direct regulation of drug-metabolizing enzymes and transporters by miRNAs; however, potential indirect regulation cannot be ruled out, since off-target effects of miRNAs have been somewhat overlooked. These artificial results from *in vitro* studies may partially explain discrepancies between *in vitro* and *in vivo* results.^303^

## Conclusions

Angiogenesis is required for tumors to grow to more than 2 mm in diameter, since the simple diffusion of nutrients and oxygen can no longer supply the rapid proliferation of cancer cells. Angiogenesis is a delicate balance between inhibitory and stimulatory factors. Inhibiting angiogenesis could be a new approach for GI cancer treatment. It is known that in primary tumors, angiogenesis is a continuous and highly intricate series of molecular events that eventually leads to the exponential growth of the tumor. Recently, substantial evidence has accumulated about the function of the miRNAs in both angiogenesis and metastasis of cancers. Studies have demonstrated that deregulation of many types of ncRNAs can affect angiogenesis and metastasis in GI cancers. Furthermore, the rapid development of different types of mimetics or antagonists of specific ncRNAs, as well as more effective delivery technologies, has raised the possibility to employ ncRNAs as targets to treat metastatic GI cancers. Direct links between the role of ncRNAs in angiogenesis and GI cancer metastasis remain to be fully determined. Therefore, ncRNA-based therapy is still not available in clinical settings. Even so, with further advances in technology, ncRNA-based therapeutic approaches will likely be applied to combat angiogenesis in GI cancer in the coming years.

## References

[1] Bray F., Ferlay J., Soerjomataram I., Siegel R.L., Torre L.A., Jemal A. (2018). Global cancer statistics 2018: GLOBOCAN estimates of incidence and mortality worldwide for 36 cancers in 185 countries. CA Cancer J. Clin..

[2] Saluja A., Maitra A. (2019). Pancreatitis and pancreatic cancer. Gastroenterology.

[3] Lasota J., Miettinen M. (2008). Clinical significance of oncogenic KIT and PDGFRA mutations in gastrointestinal stromal tumours. Histopathology.

[4] Li X., Nadauld L., Ootani A., Corney D.C., Pai R.K., Gevaert O., Cantrell M.A., Rack P.G., Neal J.T., Chan C.W.M. (2014). Oncogenic transformation of diverse gastrointestinal tissues in primary organoid culture. Nat. Med..

[5] Bang Y.J., Van Cutsem E., Feyereislova A., Chung H.C., Shen L., Sawaki A., Lordick F., Ohtsu A., Omuro Y., Satoh T. (2010). Trastuzumab in combination with chemotherapy versus chemotherapy alone for treatment of HER2-positive advanced gastric or gastro-oesophageal junction cancer (ToGA): a phase 3, open-label, randomised controlled trial. Lancet.

[6] Marano L., Chiari R., Fabozzi A., De Vita F., Boccardi V., Roviello G., Petrioli R., Marrelli D., Roviello F., Patriti A. (2015). c-Met targeting in advanced gastric cancer: An open challenge. Cancer Lett..

[7] Cunningham D., Humblet Y., Siena S., Khayat D., Bleiberg H., Santoro A., Bets D., Mueser M., Harstrick A., Verslype C. (2004). Cetuximab monotherapy and cetuximab plus irinotecan in irinotecan-refractory metastatic colorectal cancer. N. Engl. J. Med..

[8] Russo M., Crisafulli G., Sogari A., Reilly N.M., Arena S., Lamba S., Bartolini A., Amodio V., Magrì A., Novara L. (2019). Adaptive mutability of colorectal cancers in response to targeted therapies. Science.

[9] Takahashi Y., Tucker S.L., Kitadai Y., Koura A.N., Bucana C.D., Cleary K.R., Ellis L.M. (1997). Vessel counts and expression of vascular endothelial growth factor as prognostic factors in node-negative colon cancer. Arch. Surg..

[10] Takahashi Y., Kitadai Y., Bucana C.D., Cleary K.R., Ellis L.M. (1995). Expression of vascular endothelial growth factor and its receptor, KDR, correlates with vascularity, metastasis, and proliferation of human colon cancer. Cancer Res..

[11] Kang S.M., Maeda K., Onoda N., Chung Y.S., Nakata B., Nishiguchi Y., Sowa M. (1997). Combined analysis of p53 and vascular endothelial growth factor expression in colorectal carcinoma for determination of tumor vascularity and liver metastasis. Int. J. Cancer.

[12] Ishigami S.I., Arii S., Furutani M., Niwano M., Harada T., Mizumoto M., Mori A., Onodera H., Imamura M. (1998). Predictive value of vascular endothelial growth factor (VEGF) in metastasis and prognosis of human colorectal cancer. Br. J. Cancer.

[13] Ferrara N., Hillan K.J., Novotny W. (2005). Bevacizumab (Avastin), a humanized anti-VEGF monoclonal antibody for cancer therapy. Biochem. Biophys. Res. Commun..

[14] Konno S., Takebayashi Y., Aiba M., Akiyama S., Ogawa K. (2001). Clinicopathological and prognostic significance of thymidine phosphorylase and proliferating cell nuclear antigen in gastric carcinoma. Cancer Lett..

[15] Kimura H., Konishi K., Nukui T., Kaji M., Maeda K., Yabushita K., Tsuji M., Miwa A. (2001). Prognostic significance of expression of thymidine phosphorylase and vascular endothelial growth factor in human gastric carcinoma. J. Surg. Oncol..

[16] Takahashi Y., Bucana C.D., Akagi Y., Liu W., Cleary K.R., Mai M., Ellis L.M. (1998). Significance of platelet-derived endothelial cell growth factor in the angiogenesis of human gastric cancer. Clin. Cancer Res..

[17] Maeda K., Chung Y.S., Ogawa Y., Takatsuka S., Kang S.M., Ogawa M., Sawada T., Sowa M. (1996). Prognostic value of vascular endothelial growth factor expression in gastric carcinoma. Cancer.

[18] Karayiannakis A.J., Bolanaki H., Syrigos K.N., Asimakopoulos B., Polychronidis A., Anagnostoulis S., Simopoulos C. (2003). Serum vascular endothelial growth factor levels in pancreatic cancer patients correlate with advanced and metastatic disease and poor prognosis. Cancer Lett..

[19] Liu C.D., Tilch L., Kwan D., McFadden D.W. (2002). Vascular endothelial growth factor is increased in ascites from metastatic pancreatic cancer. J. Surg. Res..

[20] Niedergethmann M., Hildenbrand R., Wostbrock B., Hartel M., Sturm J.W., Richter A., Post S. (2002). High expression of vascular endothelial growth factor predicts early recurrence and poor prognosis after curative resection for ductal adenocarcinoma of the pancreas. Pancreas.

[21] Ellis L.M., Takahashi Y., Fenoglio C.J., Cleary K.R., Bucana C.D., Evans D.B. (1998). Vessel counts and vascular endothelial growth factor expression in pancreatic adenocarcinoma. Eur. J. Cancer.

[22] Ikeda N., Adachi M., Taki T., Huang C., Hashida H., Takabayashi A., Sho M., Nakajima Y., Kanehiro H., Hisanaga M. (1999). Prognostic significance of angiogenesis in human pancreatic cancer. Br. J. Cancer.

[23] Auguste P., Javerzat S., Bikfalvi A. (2003). Regulation of vascular development by fibroblast growth factors. Cell Tissue Res..

[24] Montesano R., Vassalli J.-D., Baird A., Guillemin R., Orci L. (1986). Basic fibroblast growth factor induces angiogenesis in vitro. Proc. Natl. Acad. Sci. USA.

[25] Johnson D.E., Lu J., Chen H., Werner S., Williams L.T. (1991). The human fibroblast growth factor receptor genes: a common structural arrangement underlies the mechanisms for generating receptor forms that differ in their third immunoglobulin domain. Mol. Cell. Biol..

[26] Nakamura T., Mochizuki Y., Kanetake H., Kanda S. (2001). Signals via FGF receptor 2 regulate migration of endothelial cells. Biochem. Biophys. Res. Commun..

[27] Javerzat S., Auguste P., Bikfalvi A. (2002). The role of fibroblast growth factors in vascular development. Trends Mol. Med..

[28] Arbeit J.M., Olson D.C., Hanahan D. (1996). Upregulation of fibroblast growth factors and their receptors during multi-stage epidermal carcinogenesis in K14-HPV16 transgenic mice. Oncogene.

[29] Kwan C.-P., Venkataraman G., Shriver Z., Raman R., Liu D., Qi Y., Varticovski L., Sasisekharan R. (2001). Probing fibroblast growth factor dimerization and role of heparin-like glycosaminoglycans in modulating dimerization and signaling. J. Biol. Chem..

[30] Schlessinger J., Plotnikov A.N., Ibrahimi O.A., Eliseenkova A.V., Yeh B.K., Yayon A., Linhardt R.J., Mohammadi M. (2000). Crystal structure of a ternary FGF-FGFR-heparin complex reveals a dual role for heparin in FGFR binding and dimerization. Mol. Cell.

[31] Nguyen M., Watanabe H., Budson A.E., Richie J.P., Hayes D.F., Folkman J. (1994). Elevated levels of an angiogenic peptide, basic fibroblast growth factor, in the urine of patients with a wide spectrum of cancers. J. Natl. Cancer Inst..

[32] Nguyen M., Watanabe H., Budson A.E., Richie J.P., Folkman J. (1993). Elevated levels of the angiogenic peptide basic fibroblast growth factor in urine of bladder cancer patients. J. Natl. Cancer Inst..

[33] Landriscina M., Cassano A., Ratto C., Longo R., Ippoliti M., Palazzotti B., Crucitti F., Barone C. (1998). Quantitative analysis of basic fibroblast growth factor and vascular endothelial growth factor in human colorectal cancer. Br. J. Cancer.

[34] Galzie Z., Fernig D.G., Smith J.A., Poston G.J., Kinsella A.R. (1997). Invasion of human colorectal carcinoma cells is promoted by endogenous basic fibroblast growth factor. Int. J. Cancer.

[35] George M.L., Tutton M.G., Abulafi A.M., Eccles S.A., Swift R.I. (2002). Plasma basic fibroblast growth factor levels in colorectal cancer: a clinically useful assay?. Clin. Exp. Metastasis.

[36] Grotowski M., Piechota W. (2001). Receptors of selected cytokines and angiokine bFGF in patients with colorectal cancer (a preliminary study). Pol. Merkur. Lekarski.

[37] Dirix L.Y., Vermeulen P.B., Pawinski A., Prové A., Benoy I., De Pooter C., Martin M., Van Oosterom A.T. (1997). Elevated levels of the angiogenic cytokines basic fibroblast growth factor and vascular endothelial growth factor in sera of cancer patients. Br. J. Cancer.

[38] Dirix L.Y., Vermeulen P.B., Hubens G., Benoy I., Martin M., De Pooter C., Van Oosterom A.T. (1996). Serum basic fibroblast growth factor and vascular endothelial growth factor and tumour growth kinetics in advanced colorectal cancer. Ann. Oncol..

[39] Tanimoto H., Yoshida K., Yokozaki H., Yasui W., Nakayama H., Ito H., Ohama K., Tahara E. (1991). Expression of basic fibroblast growth factor in human gastric carcinomas. Virchows Arch. B Cell Pathol. Incl. Mol. Pathol..

[40] Noda M., Hattori T., Kimura T., Naitoh H., Kodama T., Kashima K., Pignatelli M. (1997). Expression of fibroblast growth factor 2 mRNA in early and advanced gastric cancer. Acta Oncol..

[41] Ueki T., Koji T., Tamiya S., Nakane P.K., Tsuneyoshi M. (1995). Expression of basic fibroblast growth factor and fibroblast growth factor receptor in advanced gastric carcinoma. J. Pathol..

[42] Yoshikawa T., Tsuburaya A., Kobayashi O., Sairenji M., Motohashi H., Yanoma S., Noguchi Y. (2000). Plasma concentrations of VEGF and bFGF in patients with gastric carcinoma. Cancer Lett..

[43] Anzai H., Kitadai Y., Bucana C.D., Sanchez R., Omoto R., Fidler I.J. (1998). Expression of metastasis-related genes in surgical specimens of human gastric cancer can predict disease recurrence. Eur. J. Cancer.

[44] Estival A., Durand S., Clerc P., Louvel D., Vaysse N., Valdiguié P., Clemente F. (1997). Pancreatic cancer cell regulation by lipids and by basic fibroblast growth factor expression. Cancer Detect. Prev..

[45] Ghaneh P., Kawesha A., Evans J.D., Neoptolemos J.P. (2002). Molecular prognostic markers in pancreatic cancer. J. Hepatobiliary Pancreat. Surg..

[46] Fujioka S., Yoshida K., Yanagisawa S., Kawakami M., Aoki T., Yamazaki Y. (2001). Angiogenesis in pancreatic carcinoma: thymidine phosphorylase expression in stromal cells and intratumoral microvessel density as independent predictors of overall and relapse-free survival. Cancer.

[47] Ohta T., Yamamoto M., Numata M., Iseki S., Tsukioka Y., Miyashita T., Kayahara M., Nagakawa T., Miyazaki I., Nishikawa K., Yoshitake Y. (1995). Expression of basic fibroblast growth factor and its receptor in human pancreatic carcinomas. Br. J. Cancer.

[48] Jin K.-T., Yao J.-Y., Fang X.-L., Di H., Ma Y.-Y. (2020). Roles of lncRNAs in cancer: Focusing on angiogenesis. Life Sci..

[49] Cech T.R., Steitz J.A. (2014). The noncoding RNA revolution-trashing old rules to forge new ones. Cell.

[50] Peng J.-F., Zhuang Y.-Y., Huang F.-T., Zhang S.-N. (2016). Noncoding RNAs and pancreatic cancer. World J. Gastroenterol..

[51] Vorvis C., Hatziapostolou M., Mahurkar-Joshi S., Koutsioumpa M., Williams J., Donahue T.R., Poultsides G.A., Eibl G., Iliopoulos D. (2016). Transcriptomic and CRISPR/Cas9 technologies reveal FOXA2 as a tumor suppressor gene in pancreatic cancer. Am. J. Physiol. Gastrointest. Liver Physiol..

[52] Chandra Gupta S., Nandan Tripathi Y. (2017). Potential of long non-coding RNAs in cancer patients: From biomarkers to therapeutic targets. Int. J. Cancer.

[53] Bartel D.P. (2004). MicroRNAs: genomics, biogenesis, mechanism, and function. Cell.

[54] Rossbach M. (2010). Small non-coding RNAs as novel therapeutics. Curr. Mol. Med..

[55] Nelson K.M., Weiss G.J. (2008). MicroRNAs and cancer: past, present, and potential future. Mol. Cancer Ther..

[56] Macfarlane L.-A., Murphy P.R. (2010). MicroRNA: biogenesis, function and role in cancer. Curr. Genomics.

[57] Smith H.C. (2016). The role of microRNAs in gastric cancer. Am. J. Dig. Dis..

[58] Selbach M., Schwanhausser B., Thierfelder N., Fang Z., Khanin R., Rajewsky N. (2008). Widespread changes in protein synthesis induced by microRNAs. Nature.

[59] Baek D., Villén J., Shin C., Camargo F.D., Gygi S.P., Bartel D.P. (2008). The impact of microRNAs on protein output. Nature.

[60] Suzuki H.I., Yamagata K., Sugimoto K., Iwamoto T., Kato S., Miyazono K. (2009). Modulation of microRNA processing by p53. Nature.

[61] Iorio M.V., Ferracin M., Liu C.-G., Veronese A., Spizzo R., Sabbioni S., Magri E., Pedriali M., Fabbri M., Campiglio M. (2005). MicroRNA gene expression deregulation in human breast cancer. Cancer Res..

[62] Ichimi T., Enokida H., Okuno Y., Kunimoto R., Chiyomaru T., Kawamoto K., Kawahara K., Toki K., Kawakami K., Nishiyama K. (2009). Identification of novel microRNA targets based on microRNA signatures in bladder cancer. Int. J. Cancer.

[63] Xu X., Wu X., Jiang Q., Sun Y., Liu H., Chen R., Wu S. (2015). Downregulation of microRNA-1 and microRNA-145 contributes synergistically to the development of colon cancer. Int. J. Mol. Med..

[64] Cordes K.R., Sheehy N.T., White M.P., Berry E.C., Morton S.U., Muth A.N., Lee T.H., Miano J.M., Ivey K.N., Srivastava D. (2009). miR-145 and miR-143 regulate smooth muscle cell fate and plasticity. Nature.

[65] Xu Q., Liu L.-Z., Qian X., Chen Q., Jiang Y., Li D., Lai L., Jiang B.H. (2012). MiR-145 directly targets p70S6K1 in cancer cells to inhibit tumor growth and angiogenesis. Nucleic Acids Res..

[66] Lee J.-J., Drakaki A., Iliopoulos D., Struhl K. (2012). MiR-27b targets PPARγ to inhibit growth, tumor progression and the inflammatory response in neuroblastoma cells. Oncogene.

[67] Urbich C., Kaluza D., Frömel T., Knau A., Bennewitz K., Boon R.A., Bonauer A., Doebele C., Boeckel J.N., Hergenreider E. (2012). MicroRNA-27a/b controls endothelial cell repulsion and angiogenesis by targeting semaphorin 6A. Blood.

[68] Biyashev D., Veliceasa D., Topczewski J., Topczewska J.M., Mizgirev I., Vinokour E., Reddi A.L., Licht J.D., Revskoy S.Y., Volpert O.V. (2012). miR-27b controls venous specification and tip cell fate. Blood.

[69] Ye J., Wu X., Wu D., Wu P., Ni C., Zhang Z., Chen Z., Qiu F., Xu J., Huang J. (2013). miRNA-27b targets vascular endothelial growth factor C to inhibit tumor progression and angiogenesis in colorectal cancer. PLoS ONE.

[70] Xiao X., Tang C., Xiao S., Fu C., Yu P. (2013). Enhancement of proliferation and invasion by MicroRNA-590-5p via targeting PBRM1 in clear cell renal carcinoma cells. Oncol. Res..

[71] Chu Y., Ouyang Y., Wang F., Zheng A., Bai L., Han L., Chen Y., Wang H. (2014). MicroRNA-590 promotes cervical cancer cell growth and invasion by targeting CHL1. J. Cell. Biochem..

[72] Wandrey F., Montellese C., Koos K., Badertscher L., Bammert L., Cook A.G., Zemp I., Horvath P., Kutay U. (2015). The NF45/NF90 heterodimer contributes to the biogenesis of 60S ribosomal subunits and influences nucleolar morphology. Mol. Cell. Biol..

[73] Shim J., Lim H., R Yates J., Karin M. (2002). Nuclear export of NF90 is required for interleukin-2 mRNA stabilization. Mol. Cell.

[74] Patiño C., Haenni A.-L., Urcuqui-Inchima S. (2015). NF90 isoforms, a new family of cellular proteins involved in viral replication?. Biochimie.

[75] Shamanna R.A., Hoque M., Pe’ery T., Mathews M.B. (2013). Induction of p53, p21 and apoptosis by silencing the NF90/NF45 complex in human papilloma virus-transformed cervical carcinoma cells. Oncogene.

[76] Zhou Q., Zhu Y., Wei X., Zhou J., Chang L., Sui H., Han Y., Piao D., Sha R., Bai Y. (2016). MiR-590-5p inhibits colorectal cancer angiogenesis and metastasis by regulating nuclear factor 90/vascular endothelial growth factor A axis. Cell Death Dis..

[77] Yamamoto K., Ito S., Hanafusa H., Shimizu K., Ouchida M. (2015). Uncovering direct targets of miR-19a involved in lung cancer progression. PLoS ONE.

[78] Lu W.-D., Zuo Y., Xu Z., Zhang M. (2015). MiR-19a promotes epithelial-mesenchymal transition through PI3K/AKT pathway in gastric cancer. World J. Gastroenterol..

[79] Chen M., Lin M., Wang X. (2018). Overexpression of miR-19a inhibits colorectal cancer angiogenesis by suppressing KRAS expression. Oncol. Rep..

[80] Cai C., Rodepeter F.R., Rossmann A., Teymoortash A., Lee J.-S., Quint K., DI Fazio P., Ocker M., Werner J.A., Mandic R. (2012). SIVmac_239_-Nef down-regulates cell surface expression of CXCR4 in tumor cells and inhibits proliferation, migration and angiogenesis. Anticancer Res..

[81] Milligan G. (2004). G protein-coupled receptor dimerization: function and ligand pharmacology. Mol. Pharmacol..

[82] Rosenbaum D.M., Rasmussen S.G., Kobilka B.K. (2009). The structure and function of G-protein-coupled receptors. Nature.

[83] Song Z.-Y., Wang F., Cui S.-X., Qu X.-J. (2018). Knockdown of CXCR4 inhibits CXCL12-induced angiogenesis in HUVECs through downregulation of the MAPK/ERK and PI3K/AKT and the Wnt/β-catenin pathways. Cancer Invest..

[84] Fang Y., Sun B., Wang J., Wang Y. (2019). miR-622 inhibits angiogenesis by suppressing the CXCR4-VEGFA axis in colorectal cancer. Gene.

[85] Zhu D., Sun Y., Zhang D., Dong M., Jiang G., Zhang X., Zhou J. (2018). miR-1 inhibits the progression of colon cancer by regulating the expression of vascular endothelial growth factor. Oncol. Rep..

[86] Wang Y., Wu M., Lei Z., Huang M., Li Z., Wang L., Cao Q., Han D., Chang Y., Chen Y. (2018). Dysregulation of miR-6868-5p/FOXM1 circuit contributes to colorectal cancer angiogenesis. J. Exp. Clin. Cancer Res.

[87] Liang L., Gao C., Li Y., Sun M., Xu J., Li H., Jia L., Zhao Y. (2017). miR-125a-3p/FUT5-FUT6 axis mediates colorectal cancer cell proliferation, migration, invasion and pathological angiogenesis via PI3K-Akt pathway. Cell Death Dis..

[88] Ma H., Pan J.S., Jin L.X., Wu J., Ren Y.D., Chen P., Xiao C., Han J. (2016). MicroRNA-17∼92 inhibits colorectal cancer progression by targeting angiogenesis. Cancer Lett..

[89] Thuringer D., Jego G., Berthenet K., Hammann A., Solary E., Garrido C. (2016). Gap junction-mediated transfer of miR-145-5p from microvascular endothelial cells to colon cancer cells inhibits angiogenesis. Oncotarget.

[90] Zhang Y., Wang X., Xu B., Wang B., Wang Z., Liang Y., Zhou J., Hu J., Jiang B. (2013). Epigenetic silencing of miR-126 contributes to tumor invasion and angiogenesis in colorectal cancer. Oncol. Rep..

[91] Xiao F., Qiu H., Cui H., Ni X., Li J., Liao W., Lu L., Ding K. (2015). MicroRNA-885-3p inhibits the growth of HT-29 colon cancer cell xenografts by disrupting angiogenesis via targeting BMPR1A and blocking BMP/Smad/Id1 signaling. Oncogene.

[92] Chen X., Xu X., Pan B., Zeng K., Xu M., Liu X., He B., Pan Y., Sun H., Wang S. (2018). miR-150-5p suppresses tumor progression by targeting VEGFA in colorectal cancer. Aging (Albany NY).

[93] Qian X., Yu J., Yin Y., He J., Wang L., Li Q., Zhang L.Q., Li C.Y., Shi Z.M., Xu Q. (2013). MicroRNA-143 inhibits tumor growth and angiogenesis and sensitizes chemosensitivity to oxaliplatin in colorectal cancers. Cell Cycle.

[94] Yamakuchi M., Lotterman C.D., Bao C., Hruban R.H., Karim B., Mendell J.T., Huso D., Lowenstein C.J. (2010). P53-induced microRNA-107 inhibits HIF-1 and tumor angiogenesis. Proc. Natl. Acad. Sci. USA.

[95] Li Y., Kuscu C., Banach A., Zhang Q., Pulkoski-Gross A., Kim D., Liu J., Roth E., Li E., Shroyer K.R. (2015). miR-181a-5p inhibits cancer cell migration and angiogenesis via downregulation of matrix metalloproteinase-14. Cancer Res..

[96] Wei L., Sun C., Zhang Y., Han N., Sun S. (2020). miR-503-5p inhibits colon cancer tumorigenesis, angiogenesis, and lymphangiogenesis by directly downregulating VEGF-A. Gene Ther..

[97] Yan S., Wang H., Chen X., Liang C., Shang W., Wang L., Li J., Xu D. (2020). MiR-182-5p inhibits colon cancer tumorigenesis, angiogenesis, and lymphangiogenesis by directly downregulating VEGF-C. Cancer Lett..

[98] Li X., Li Z., Zhu Y., Li Z., Yao L., Zhang L., Yuan L., Shang Y., Liu J., Li C. (2020). miR-524-5p inhibits angiogenesis through targeting WNK1 in colon cancer cells. Am. J. Physiol. Gastrointest. Liver Physiol..

[99] Luo H.-N., Wang Z.-H., Sheng Y., Zhang Q., Yan J., Hou J., Zhu K., Cheng Y., Xu Y.L., Zhang X.H. (2014). MiR-139 targets CXCR4 and inhibits the proliferation and metastasis of laryngeal squamous carcinoma cells. Med. Oncol..

[100] Li L., Li B., Chen D., Liu L., Huang C., Lu Z., Lun L., Wan X. (2015). miR-139 and miR-200c regulate pancreatic cancer endothelial cell migration and angiogenesis. Oncol. Rep..

[101] Boise L.H., González-García M., Postema C.E., Ding L., Lindsten T., Turka L.A., Mao X., Nuñez G., Thompson C.B. (1993). bcl-x, a bcl-2-related gene that functions as a dominant regulator of apoptotic cell death. Cell.

[102] Wei M.C., Zong W.-X., Cheng E.H.-Y., Lindsten T., Panoutsakopoulou V., Ross A.J., Roth K.A., MacGregor G.R., Thompson C.B., Korsmeyer S.J. (2001). Proapoptotic BAX and BAK: a requisite gateway to mitochondrial dysfunction and death. Science.

[103] Liu R., Zhang H., Wang X., Zhou L., Li H., Deng T., Qu Y., Duan J., Bai M., Ge S. (2015). The miR-24-Bim pathway promotes tumor growth and angiogenesis in pancreatic carcinoma. Oncotarget.

[104] Park Y.-A., Choi C.H., Do I.-G., Song S.Y., Lee J.K., Cho Y.J., Choi J.J., Jeon H.K., Ryu J.Y., Lee Y.Y. (2014). Dual targeting of angiotensin receptors (AGTR1 and AGTR2) in epithelial ovarian carcinoma. Gynecol. Oncol..

[105] Shibata K., Kikkawa F., Mizokami Y., Kajiyama H., Ino K., Nomura S., Mizutani S. (2005). Possible involvement of adipocyte-derived leucine aminopeptidase via angiotensin II in endometrial carcinoma. Tumour Biol..

[106] Watanabe Y., Shibata K., Kikkawa F., Kajiyama H., Ino K., Hattori A., Tsujimoto M., Mizutani S. (2003). Adipocyte-derived leucine aminopeptidase suppresses angiogenesis in human endometrial carcinoma via renin-angiotensin system. Clin. Cancer Res..

[107] Du N., Feng J., Hu L.-J., Sun X., Sun H.-B., Zhao Y., Yang Y.P., Ren H. (2012). Angiotensin II receptor type 1 blockers suppress the cell proliferation effects of angiotensin II in breast cancer cells by inhibiting AT1R signaling. Oncol. Rep..

[108] Chen X., Meng Q., Zhao Y., Liu M., Li D., Yang Y., Sun L., Sui G., Cai L., Dong X. (2013). Angiotensin II type 1 receptor antagonists inhibit cell proliferation and angiogenesis in breast cancer. Cancer Lett..

[109] Guo R., Gu J., Zhang Z., Wang Y., Gu C. (2015). MicroRNA-410 functions as a tumor suppressor by targeting angiotensin II type 1 receptor in pancreatic cancer. IUBMB Life.

[110] Zhang J.-G., Farley A., Nicholson S.E., Willson T.A., Zugaro L.M., Simpson R.J., Moritz R.L., Cary D., Richardson R., Hausmann G. (1999). The conserved SOCS box motif in suppressors of cytokine signaling binds to elongins B and C and may couple bound proteins to proteasomal degradation. Proc. Natl. Acad. Sci. USA.

[111] Nicholson S.E., Hilton D.J. (1998). The SOCS proteins: a new family of negative regulators of signal transduction. J. Leukoc. Biol..

[112] Subramanian A., Kuehn H., Gould J., Tamayo P., Mesirov J.P. (2007). GSEA-P: a desktop application for Gene Set Enrichment Analysis. Bioinformatics.

[113] Feng Z.P., Chandrashekaran I.R., Low A., Speed T.P., Nicholson S.E., Norton R.S. (2012). The N-terminal domains of SOCS proteins: a conserved region in the disordered N-termini of SOCS4 and 5. Proteins.

[114] Hu H., Zhang Q., Chen W., Wu T., Liu S., Li X., Luo B., Zhang T., Yan G., Lu H., Lu Z. (2020). MicroRNA-301a promotes pancreatic cancer invasion and metastasis through the JAK/STAT3 signaling pathway by targeting SOCS5. Carcinogenesis.

[115] Sun L., Wang Q., Gao X., Shi D., Mi S., Han Q. (2015). MicroRNA-454 functions as an oncogene by regulating PTEN in uveal melanoma. FEBS Lett..

[116] Zhu D.-Y., Li X.-N., Qi Y., Liu D.-L., Yang Y., Zhao J., Zhang C.Y., Wu K., Zhao S. (2016). MiR-454 promotes the progression of human non-small cell lung cancer and directly targets PTEN. Biomed. Pharmacother..

[117] Fan Y., Shi C., Li T., Kuang T. (2017). microRNA-454 shows anti-angiogenic and anti-metastatic activity in pancreatic ductal adenocarcinoma by targeting LRP6. Am. J. Cancer Res..

[118] Xiong Y., Fang J.H., Yun J.P., Yang J., Zhang Y., Jia W.H., Zhuang S.M. (2010). Effects of microRNA-29 on apoptosis, tumorigenicity, and prognosis of hepatocellular carcinoma. Hepatology.

[119] Cortez M.A., Nicoloso M.S., Shimizu M., Rossi S., Gopisetty G., Molina J.R., Carlotti C., Tirapelli D., Neder L., Brassesco M.S. (2010). miR-29b and miR-125a regulate podoplanin and suppress invasion in glioblastoma. Genes Chromosomes Cancer.

[120] Muniyappa M.K., Dowling P., Henry M., Meleady P., Doolan P., Gammell P., Clynes M., Barron N. (2009). MiRNA-29a regulates the expression of numerous proteins and reduces the invasiveness and proliferation of human carcinoma cell lines. Eur. J. Cancer.

[121] Zhao J.-J., Lin J., Lwin T., Yang H., Guo J., Kong W., Dessureault S., Moscinski L.C., Rezania D., Dalton W.S. (2010). microRNA expression profile and identification of miR-29 as a prognostic marker and pathogenetic factor by targeting CDK6 in mantle cell lymphoma. Blood.

[122] Kessenbrock K., Plaks V., Werb Z. (2010). Matrix metalloproteinases: regulators of the tumor microenvironment. Cell.

[123] Ogasawara S., Yano H., Momosaki S., Nishida N., Takemoto Y., Kojiro S., Kojiro M. (2005). Expression of matrix metalloproteinases (MMPs) in cultured hepatocellular carcinoma (HCC) cells and surgically resected HCC tissues. Oncol. Rep..

[124] Giannelli G., Bergamini C., Marinosci F., Fransvea E., Quaranta M., Lupo L., Schiraldi O., Antonaci S. (2002). Clinical role of MMP-2/TIMP-2 imbalance in hepatocellular carcinoma. Int. J. Cancer.

[125] Fang J.H., Zhou H.C., Zeng C., Yang J., Liu Y., Huang X., Zhang J.P., Guan X.Y., Zhuang S.M. (2011). MicroRNA-29b suppresses tumor angiogenesis, invasion, and metastasis by regulating matrix metalloproteinase 2 expression. Hepatology.

[126] Bentwich I., Avniel A., Karov Y., Aharonov R., Gilad S., Barad O., Barzilai A., Einat P., Einav U., Meiri E. (2005). Identification of hundreds of conserved and nonconserved human microRNAs. Nat. Genet..

[127] Sewer A., Paul N., Landgraf P., Aravin A., Pfeffer S., Brownstein M.J., Tuschl T., van Nimwegen E., Zavolan M. (2005). Identification of clustered microRNAs using an ab initio prediction method. BMC Bioinformatics.

[128] Landgraf P., Rusu M., Sheridan R., Sewer A., Iovino N., Aravin A., Pfeffer S., Rice A., Kamphorst A.O., Landthaler M. (2007). A mammalian microRNA expression atlas based on small RNA library sequencing. Cell.

[129] Caporali A., Meloni M., Völlenkle C., Bonci D., Sala-Newby G.B., Addis R., Spinetti G., Losa S., Masson R., Baker A.H. (2011). Deregulation of microRNA-503 contributes to diabetes mellitus-induced impairment of endothelial function and reparative angiogenesis after limb ischemia. Circulation.

[130] Zhou B., Ma R., Si W., Li S., Xu Y., Tu X., Wang Q. (2013). MicroRNA-503 targets FGF2 and VEGFA and inhibits tumor angiogenesis and growth. Cancer Lett..

[131] Rosa R., Marciano R., Malapelle U., Formisano L., Nappi L., D’Amato C., D’Amato V., Damiano V., Marfè G., Del Vecchio S. (2013). Sphingosine kinase 1 overexpression contributes to cetuximab resistance in human colorectal cancer models. Clin. Cancer Res..

[132] Nagahashi M., Ramachandran S., Kim E.Y., Allegood J.C., Rashid O.M., Yamada A., Zhao R., Milstien S., Zhou H., Spiegel S., Takabe K. (2012). Sphingosine-1-phosphate produced by sphingosine kinase 1 promotes breast cancer progression by stimulating angiogenesis and lymphangiogenesis. Cancer Res..

[133] Yang L., Yue S., Yang L., Liu X., Han Z., Zhang Y., Li L. (2013). Sphingosine kinase/sphingosine 1-phosphate (S1P)/S1P receptor axis is involved in liver fibrosis-associated angiogenesis. J. Hepatol..

[134] Lu Z., Zhang W., Gao S., Jiang Q., Xiao Z., Ye L., Zhang X. (2015). MiR-506 suppresses liver cancer angiogenesis through targeting sphingosine kinase 1 (SPHK1) mRNA. Biochem. Biophys. Res. Commun..

[135] Curtale G., Citarella F., Carissimi C., Goldoni M., Carucci N., Fulci V., Franceschini D., Meloni F., Barnaba V., Macino G. (2010). An emerging player in the adaptive immune response: microRNA-146a is a modulator of IL-2 expression and activation-induced cell death in T lymphocytes. Blood.

[136] He H., Jazdzewski K., Li W., Liyanarachchi S., Nagy R., Volinia S., Calin G.A., Liu C.G., Franssila K., Suster S. (2005). The role of microRNA genes in papillary thyroid carcinoma. Proc. Natl. Acad. Sci. USA.

[137] Bhaumik D., Scott G.K., Schokrpur S., Patil C.K., Campisi J., Benz C.C. (2008). Expression of microRNA-146 suppresses NF-kappaB activity with reduction of metastatic potential in breast cancer cells. Oncogene.

[138] Hurst D.R., Edmonds M.D., Scott G.K., Benz C.C., Vaidya K.S., Welch D.R. (2009). Breast cancer metastasis suppressor 1 up-regulates miR-146, which suppresses breast cancer metastasis. Cancer Res..

[139] Li Y., Vandenboom T.G., Wang Z., Kong D., Ali S., Philip P.A., Sarkar F.H. (2010). miR-146a suppresses invasion of pancreatic cancer cells. Cancer Res..

[140] Wang X., Tang S., Le S.-Y., Lu R., Rader J.S., Meyers C., Zheng Z.M. (2008). Aberrant expression of oncogenic and tumor-suppressive microRNAs in cervical cancer is required for cancer cell growth. PLoS ONE.

[141] Zhu K., Pan Q., Zhang X., Kong L.-Q., Fan J., Dai Z., Wang L., Yang X.R., Hu J., Wan J.L. (2013). MiR-146a enhances angiogenic activity of endothelial cells in hepatocellular carcinoma by promoting PDGFRA expression. Carcinogenesis.

[142] Ebrahimi F., Gopalan V., Smith R.A., Lam A.K.-Y. (2014). miR-126 in human cancers: clinical roles and current perspectives. Exp. Mol. Pathol..

[143] Wang S., Wang X., Guo Q., Wang G., Han X., Li X., Shi Z.W., He W. (2016). MicroRNA-126 overexpression inhibits proliferation and invasion in osteosarcoma cells. Technol. Cancer Res. Treat..

[144] Guo C., Sah J.F., Beard L., Willson J.K., Markowitz S.D., Guda K. (2008). The noncoding RNA, miR-126, suppresses the growth of neoplastic cells by targeting phosphatidylinositol 3-kinase signaling and is frequently lost in colon cancers. Genes Chromosomes Cancer.

[145] Zhao C., Li Y., Zhang M., Yang Y., Chang L. (2015). miR-126 inhibits cell proliferation and induces cell apoptosis of hepatocellular carcinoma cells partially by targeting Sox2. Hum. Cell.

[146] Gong C., Fang J., Li G., Liu H.-H., Liu Z.-S. (2017). Effects of microRNA-126 on cell proliferation, apoptosis and tumor angiogenesis via the down-regulating ERK signaling pathway by targeting EGFL7 in hepatocellular carcinoma. Oncotarget.

[147] Yang C., Xu Y., Cheng F., Hu Y., Yang S., Rao J., Wang X. (2017). miR-1301 inhibits hepatocellular carcinoma cell migration, invasion, and angiogenesis by decreasing Wnt/β-catenin signaling through targeting BCL9. Cell Death Dis..

[148] Wang Y., Sun B., Sun H., Zhao X., Wang X., Zhao N., Zhang Y., Li Y., Gu Q., Liu F. (2016). Regulation of proliferation, angiogenesis and apoptosis in hepatocellular carcinoma by miR-26b-5p. Tumour Biol..

[149] Ghosh A., Dasgupta D., Ghosh A., Roychoudhury S., Kumar D., Gorain M., Butti R., Datta S., Agarwal S., Gupta S. (2017). MiRNA199a-3p suppresses tumor growth, migration, invasion and angiogenesis in hepatocellular carcinoma by targeting VEGFA, VEGFR1, VEGFR2, HGF and MMP2. Cell Death Dis..

[150] Wu M., Huang C., Huang X., Liang R., Feng Y., Luo X. (2017). MicroRNA-144-3p suppresses tumor growth and angiogenesis by targeting SGK3 in hepatocellular carcinoma. Oncol. Rep..

[151] Yan J.J., Zhang Y.N., Liao J.Z., Ke K.P., Chang Y., Li P.Y., Wang M., Lin J.S., He X.X. (2015). MiR-497 suppresses angiogenesis and metastasis of hepatocellular carcinoma by inhibiting VEGFA and AEG-1. Oncotarget.

[152] Yu Q., Xiang L., Yin L., Liu X., Yang D. and Zhou, J. (2017). Loss-of-function of miR-142 by hypermethylation promotes TGF-β-mediated tumour growth and metastasis in hepatocellular carcinoma. Cell Prolif.

[153] Zhang T., Liu W., Zeng X.C., Jiang N., Fu B.S., Guo Y., Yi H.M., Li H., Zhang Q., Chen W.J., Chen G.H. (2016). Down-regulation of microRNA-338-3p promoted angiogenesis in hepatocellular carcinoma. Biomed. Pharmacother..

[154] Cheng J., Chen Y., Zhao P., Liu X., Dong J., Li J., Huang C., Wu R., Lv Y. (2016). Downregulation of miRNA-638 promotes angiogenesis and growth of hepatocellular carcinoma by targeting VEGF. Oncotarget.

[155] Yang Y., Zhang J., Xia T., Li G., Tian T., Wang M., Wang R., Zhao L., Yang Y., Lan K., Zhou W. (2016). MicroRNA-210 promotes cancer angiogenesis by targeting fibroblast growth factor receptor-like 1 in hepatocellular carcinoma. Oncol. Rep..

[156] Du C., Weng X., Hu W., Lv Z., Xiao H., Ding C., Gyabaah O.A.K., Xie H., Zhou L., Wu J., Zheng S. (2015). Hypoxia-inducible MiR-182 promotes angiogenesis by targeting RASA1 in hepatocellular carcinoma. J. Exp. Clin. Cancer Res..

[157] Ji J.S., Xu M., Song J.J., Zhao Z.W., Chen M.J., Chen W.Q., Tu J.F., Yang X.M. (2016). Inhibition of microRNA-126 promotes the expression of Spred1 to inhibit angiogenesis in hepatocellular carcinoma after transcatheter arterial chemoembolization: in vivo study. OncoTargets Ther..

[158] Yang X., Zhang X.F., Lu X., Jia H.L., Liang L., Dong Q.Z., Ye Q.H., Qin L.X. (2014). MicroRNA-26a suppresses angiogenesis in human hepatocellular carcinoma by targeting hepatocyte growth factor-cMet pathway. Hepatology.

[159] Du C., Lv Z., Cao L., Ding C., Gyabaah O.A., Xie H., Zhou L., Wu J., Zheng S. (2014). MiR-126-3p suppresses tumor metastasis and angiogenesis of hepatocellular carcinoma by targeting LRP6 and PIK3R2. J. Transl. Med..

[160] Wang R., Zhao N., Li S., Fang J.H., Chen M.X., Yang J., Jia W.H., Yuan Y., Zhuang S.M. (2013). MicroRNA-195 suppresses angiogenesis and metastasis of hepatocellular carcinoma by inhibiting the expression of VEGF, VAV2, and CDC42. Hepatology.

[161] Cao Y.P., Pan M., Song Y.L., Zhang H.L., Sui H.T., Shan B.C., Piao H.X. (2019). MiR-302 a/b/c suppresses tumor angiogenesis in hepatocellular carcinoma by targeting MACC1. Eur. Rev. Med. Pharmacol. Sci..

[162] Chai Z.T., Kong J., Zhu X.D., Zhang Y.Y., Lu L., Zhou J.M., Wang L.R., Zhang K.Z., Zhang Q.B., Ao J.Y. (2013). MicroRNA-26a inhibits angiogenesis by down-regulating VEGFA through the PIK3C2α/Akt/HIF-1α pathway in hepatocellular carcinoma. PLoS ONE.

[163] Yahya S.M.M., Yahya S.M.M. (2020). The Effect of miR-98 and miR-214 on Apoptotic and Angiogenic Pathways in Hepatocellular Carcinoma HepG2 Cells. Indian J. Clin. Biochem..

[164] Li D., Wang T., Sun F.F., Feng J.Q., Peng J.J., Li H., Wang C., Wang D., Liu Y., Bai Y.D. (2020). MicroRNA-375 represses tumor angiogenesis and reverses resistance to sorafenib in hepatocarcinoma. Cancer Gene. Ther.

[165] Moh-Moh-Aung A., Fujisawa M., Ito S., Katayama H., Ohara T., Ota Y., Yoshimura T., Matsukawa A. (2020). Decreased miR-200b-3p in cancer cells leads to angiogenesis in HCC by enhancing endothelial ERG expression. Sci. Rep..

[166] O’Day E., Lal A. (2010). MicroRNAs and their target gene networks in breast cancer. Breast Cancer Res..

[167] Nam E.J., Yoon H., Kim S.W., Kim H., Kim Y.T., Kim J.H., Kim J.W., Kim S. (2008). MicroRNA expression profiles in serous ovarian carcinoma. Clin. Cancer Res..

[168] Jiang L., Huang Q., Zhang S., Zhang Q., Chang J., Qiu X., Wang E. (2010). Hsa-miR-125a-3p and hsa-miR-125a-5p are downregulated in non-small cell lung cancer and have inverse effects on invasion and migration of lung cancer cells. BMC Cancer.

[169] Ferretti E., De Smaele E., Po A., Di Marcotullio L., Tosi E., Espinola M.S.B., Di Rocco C., Riccardi R., Giangaspero F., Farcomeni A. (2009). MicroRNA profiling in human medulloblastoma. Int. J. Cancer.

[170] Hashiguchi Y., Nishida N., Mimori K., Sudo T., Tanaka F., Shibata K., Ishii H., Mochizuki H., Hase K., Doki Y., Mori M. (2012). Down-regulation of miR-125a-3p in human gastric cancer and its clinicopathological significance. Int. J. Oncol..

[171] Dai J., Wang J., Yang L., Xiao Y., Ruan Q. (2015). miR-125a regulates angiogenesis of gastric cancer by targeting vascular endothelial growth factor A. Int. J. Oncol..

[172] Jüttner S., Wissmann C., Jöns T., Vieth M., Hertel J., Gretschel S., Schlag P.M., Kemmner W., Höcker M. (2006). Vascular endothelial growth factor-D and its receptor VEGFR-3: two novel independent prognostic markers in gastric adenocarcinoma. J. Clin. Oncol..

[173] Suzuki S., Dobashi Y., Hatakeyama Y., Tajiri R., Fujimura T., Heldin C.H., Ooi A. (2010). Clinicopathological significance of platelet-derived growth factor (PDGF)-B and vascular endothelial growth factor-A expression, PDGF receptor-β phosphorylation, and microvessel density in gastric cancer. BMC Cancer.

[174] Xie M., Dart D.A., Guo T., Xing X.-F., Cheng X.-J., Du H., Jiang W.G., Wen X.Z., Ji J.F. (2018). MicroRNA-1 acts as a tumor suppressor microRNA by inhibiting angiogenesis-related growth factors in human gastric cancer. Gastric Cancer.

[175] Will M., Qin A.C.R., Toy W., Yao Z., Rodrik-Outmezguine V., Schneider C., Huang X., Monian P., Jiang X., de Stanchina E. (2014). Rapid induction of apoptosis by PI3K inhibitors is dependent upon their transient inhibition of RAS-ERK signaling. Cancer Discov..

[176] Wu Z.-H., Lin C., Liu C.-C., Jiang W.-W., Huang M.-Z., Liu X., Guo W.J. (2018). MiR-616-3p promotes angiogenesis and EMT in gastric cancer via the PTEN/AKT/mTOR pathway. Biochem. Biophys. Res. Commun..

[177] Taupin D., Podolsky D.K. (2003). Trefoil factors: initiators of mucosal healing. Nat. Rev. Mol. Cell Biol..

[178] Kim J.H., Kim M.A., Lee H.S., Kim W.H. (2009). Comparative analysis of protein expressions in primary and metastatic gastric carcinomas. Hum. Pathol..

[179] Shi Y., Huang X., Chen G., Wang Y., Liu Y., Xu W., Tang S., Guleng B., Liu J., Ren J. (2019). miR-632 promotes gastric cancer progression by accelerating angiogenesis in a TFF1-dependent manner. BMC Cancer.

[180] Li Q.-L., Ito K., Sakakura C., Fukamachi H., Inoue K.-i., Chi X.-Z., Lee K.Y., Nomura S., Lee C.W., Han S.B. (2002). Causal relationship between the loss of RUNX3 expression and gastric cancer. Cell.

[181] Lee S.H., Jung Y.D., Choi Y.S., Lee Y.M. (2015). Targeting of RUNX3 by miR-130a and miR-495 cooperatively increases cell proliferation and tumor angiogenesis in gastric cancer cells. Oncotarget.

[182] Wang L., Bo X., Zheng Q., Ge W., Liu Y., Li B. (2018). Paired box 8 suppresses tumor angiogenesis and metastasis in gastric cancer through repression of FOXM1 via induction of microRNA-612. J. Exp. Clin. Cancer Res..

[183] Zhang J.X., Chen Z.H., Chen D.L., Tian X.P., Wang C.Y., Zhou Z.W., Gao Y., Xu Y., Chen C., Zheng Z.S. (2018). LINC01410-miR-532-NCF2-NF-kB feedback loop promotes gastric cancer angiogenesis and metastasis. Oncogene.

[184] Si Y., Zhang H., Ning T., Bai M., Wang Y., Yang H., Wang X., Li J., Ying G., Ba Y. (2017). miR-26a/b Inhibit Tumor Growth and Angiogenesis by Targeting the HGF-VEGF Axis in Gastric Carcinoma. Cell Physiol. Biochem.

[185] Xie M., Dart D.A., Guo T., Xing X.F., Cheng X.J., Du H., Jiang W.G., Wen X.Z., Ji J.F. (2018). MicroRNA-1 acts as a tumor suppressor microRNA by inhibiting angiogenesis-related growth factors in human gastric cancer. Gastric Cancer.

[186] Jia L., Chen J., Xie C., Shao L., Xu Z., Zhang L. (2017). microRNA-1228^⁎^ impairs the pro-angiogenic activity of gastric cancer cells by targeting macrophage migration inhibitory factor. Life Sci..

[187] Zhang X., Dong J., He Y., Zhao M., Liu Z., Wang N., Jiang M., Zhang Z., Liu G., Liu H. (2017). miR-218 inhibited tumor angiogenesis by targeting ROBO1 in gastric cancer. Gene.

[188] Li S., Zhang H., Ning T., Wang X., Liu R., Yang H., Han Y., Deng T., Zhou L., Zhang L. (2016). MiR-520b/e Regulates Proliferation and Migration by Simultaneously Targeting EGFR in Gastric Cancer. Cell Physiol. Biochem.

[189] Liu H.T., Xing A.Y., Chen X., Ma R.R., Wang Y.W., Shi D.B., Zhang H., Li P., Chen H.F., Li Y.H., Gao P. (2015). MicroRNA-27b, microRNA-101 and microRNA-128 inhibit angiogenesis by down-regulating vascular endothelial growth factor C expression in gastric cancers. Oncotarget.

[190] Li Z., Liu Z., Dong S., Zhang J., Tan J., Wang Y., Ge C., Li R., Xue Y., Li M. (2015). miR-506 Inhibits Epithelial-to-Mesenchymal Transition and Angiogenesis in Gastric Cancer. Am. J. Pathol..

[191] Zhang X., Tang J., Zhi X., Xie K., Wang W., Li Z., Zhu Y., Yang L., Xu H., Xu Z. (2015). miR-874 functions as a tumor suppressor by inhibiting angiogenesis through STAT3/VEGF-A pathway in gastric cancer. Oncotarget.

[192] Chen H., Li L., Wang S., Lei Y., Ge Q., Lv N., Zhou X., Chen C. (2014). Reduced miR-126 expression facilitates angiogenesis of gastric cancer through its regulation on VEGF-A. Oncotarget.

[193] Zhang H., Bai M., Deng T., Liu R., Wang X., Qu Y., Duan J., Zhang L., Ning T., Ge S. (2016). Cell-derived microvesicles mediate the delivery of miR-29a/c to suppress angiogenesis in gastric carcinoma. Cancer Lett..

[194] Cheng Z., Liu F., Zhang H., Li X., Li Y., Li J., Liu F., Cao Y., Cao L., Li F. (2017). miR-135a inhibits tumor metastasis and angiogenesis by targeting FAK pathway. Oncotarget.

[195] Zheng L., Pu J., Qi T., Qi M., Li D., Xiang X., Huang K., Tong Q. (2013). miRNA-145 targets v-ets erythroblastosis virus E26 oncogene homolog 1 to suppress the invasion, metastasis, and angiogenesis of gastric cancer cells. Mol. Cancer Res..

[196] Ihara Y., Kato Y., Bando T., Yamagishi F., Minamimura T., Sakamoto T., Tsukada K., Isobe M. (2002). Allelic imbalance of 14q32 in esophageal carcinoma. Cancer Genet. Cytogenet..

[197] Haller F., Von Heydebreck A., Zhang J.D., Gunawan B., Langer C., Ramadori G., Wiemann S., Sahin O. (2010). Localization-and mutation-dependent microRNA (miRNA) expression signatures in gastrointestinal stromal tumours (GISTs), with a cluster of co-expressed miRNAs located at 14q32.31. J. Pathol.

[198] Valent P., Bonnet D., De Maria R., Lapidot T., Copland M., Melo J.V., Chomienne C., Ishikawa F., Schuringa J.J., Stassi G. (2012). Cancer stem cell definitions and terminology: the devil is in the details. Nat. Rev. Cancer.

[199] Hang D., Dong H.C., Ning T., Dong B., Hou D.L., Xu W.G. (2012). Prognostic value of the stem cell markers CD133 and ABCG2 expression in esophageal squamous cell carcinoma. Dis. Esophagus.

[200] Nakajima T.E., Yoshida H., Okamoto N., Nagashima K., Taniguchi H., Yamada Y., Shimoda T., Masutomi K. (2012). Nucleostemin and TWIST as predictive markers for recurrence after neoadjuvant chemotherapy for esophageal carcinoma. Cancer Sci..

[201] Lu C., Xu F., Gu J., Yuan Y., Zhao G., Yu X., Ge D. (2015). Clinical and biological significance of stem-like CD133(+)CXCR4(+) cells in esophageal squamous cell carcinoma. J. Thorac. Cardiovasc. Surg..

[202] Okamoto K., Ninomiya I., Ohbatake Y., Hirose A., Tsukada T., Nakanuma S., Sakai S., Kinoshita J., Makino I., Nakamura K. (2016). Expression status of CD44 and CD133 as a prognostic marker in esophageal squamous cell carcinoma treated with neoadjuvant chemotherapy followed by radical esophagectomy. Oncol. Rep..

[203] Li B., Xu W.W., Han L., Chan K.T., Tsao S.W., Lee N.P.Y., Law S., Xu L.Y., Li E.M., Chan K.W. (2017). MicroRNA-377 suppresses initiation and progression of esophageal cancer by inhibiting CD133 and VEGF. Oncogene.

[204] Liu X., Gong J., Xu B. (2015). miR-143 down-regulates TLR2 expression in hepatoma cells and inhibits hepatoma cell proliferation and invasion. Int. J. Clin. Exp. Pathol..

[205] Su J., Liang H., Yao W., Wang N., Zhang S., Yan X., Feng H., Pang W., Wang Y., Wang X. (2014). MiR-143 and MiR-145 regulate IGF1R to suppress cell proliferation in colorectal cancer. PLoS ONE.

[206] Wei J., Ma Z., Li Y., Zhao B., Wang D., Jin Y., Jin Y. (2015). miR-143 inhibits cell proliferation by targeting autophagy-related 2B in non-small cell lung cancer H1299 cells. Mol. Med. Rep..

[207] Wu D., Huang P., Wang L., Zhou Y., Pan H., Qu P. (2013). MicroRNA-143 inhibits cell migration and invasion by targeting matrix metalloproteinase 13 in prostate cancer. Mol. Med. Rep..

[208] Hicklin D.J., Ellis L.M. (2005). Role of the vascular endothelial growth factor pathway in tumor growth and angiogenesis. J. Clin. Oncol..

[209] De Falco S. (2012). The discovery of placenta growth factor and its biological activity. Exp. Mol. Med..

[210] Lee J.-M., Cimino-Mathews A., Peer C.J., Zimmer A., Lipkowitz S., Annunziata C.M., Cao L., Harrell M.I., Swisher E.M., Houston N. (2017). Safety and clinical activity of the programmed death-ligand 1 inhibitor durvalumab in combination with poly (ADP-ribose) polymerase inhibitor olaparib or vascular endothelial growth factor receptor 1-3 inhibitor cediranib in women’s cancers: a dose-escalation, phase I study. J. Clin. Oncol..

[211] Faivre S., Niccoli P., Castellano D., Valle J.W., Hammel P., Raoul J.-L., Vinik A., Van Cutsem E., Bang Y.J., Lee S.H. (2017). Sunitinib in pancreatic neuroendocrine tumors: updated progression-free survival and final overall survival from a phase III randomized study. Ann. Oncol..

[212] Hatem R., Labiod D., Château-Joubert S., de Plater L., El Botty R., Vacher S., Bonin F., Servely J.L., Dieras V., Bièche I., Marangoni E. (2016). Vandetanib as a potential new treatment for estrogen receptor-negative breast cancers. Int. J. Cancer.

[213] Jin Y.P., Hu Y.P., Wu X.S., Wu Y.S., Ye Y.Y., Li H.F., Liu Y.C., Jiang L., Liu F.T., Zhang Y.J. (2018). miR-143-3p targeting of ITGA6 suppresses tumour growth and angiogenesis by downregulating PLGF expression via the PI3K/AKT pathway in gallbladder carcinoma. Cell Death Dis..

[214] Jia H., Osak M., Bogu G.K., Stanton L.W., Johnson R., Lipovich L. (2010). Genome-wide computational identification and manual annotation of human long noncoding RNA genes. RNA.

[215] Cabili M.N., Trapnell C., Goff L., Koziol M., Tazon-Vega B., Regev A., Rinn J.L. (2011). Integrative annotation of human large intergenic noncoding RNAs reveals global properties and specific subclasses. Genes Dev..

[216] Hombach S., Kretz M. (2016). Non-coding RNAs: classification, biology and functioning. Adv. Exp. Med. Biol.

[217] Chen L.-L. (2016). Linking long noncoding RNA localization and function. Trends Biochem. Sci..

[218] Jarroux J., Morillon A., Pinskaya M. (2017). History, discovery, and classification of lncRNAs. Adv. Exp. Med. Biol.

[219] Kashi K., Henderson L., Bonetti A., Carninci P. (2016). Discovery and functional analysis of lncRNAs: Methodologies to investigate an uncharacterized transcriptome. Biochim. Biophys. Acta.

[220] Spurlock C.F., Crooke P.S., Aune T.M. (2016). Biogenesis and transcriptional regulation of long noncoding RNAs in the human immune system. J. Immunol..

[221] Clark M.B., Amaral P.P., Schlesinger F.J., Dinger M.E., Taft R.J., Rinn J.L., Ponting C.P., Stadler P.F., Morris K.V., Morillon A. (2011). The reality of pervasive transcription. PLoS Biol..

[222] Hangauer M.J., Vaughn I.W., McManus M.T. (2013). Pervasive transcription of the human genome produces thousands of previously unidentified long intergenic noncoding RNAs. PLoS Genet..

[223] Yu A.D., Wang Z., Morris K.V. (2015). Long noncoding RNAs: a potent source of regulation in immunity and disease. Immunol. Cell Biol..

[224] Khachane A.N., Harrison P.M. (2010). Mining mammalian transcript data for functional long non-coding RNAs. PLoS ONE.

[225] Moran V.A., Perera R.J., Khalil A.M. (2012). Emerging functional and mechanistic paradigms of mammalian long non-coding RNAs. Nucleic Acids Res..

[226] Aune T.M., Crooke P.S., Spurlock C.F. (2016). Long noncoding RNAs in T lymphocytes. J. Leukoc. Biol..

[227] Mattick J.S., Rinn J.L. (2015). Discovery and annotation of long noncoding RNAs. Nat. Struct. Mol. Biol..

[228] Guttman M., Rinn J.L. (2012). Modular regulatory principles of large non-coding RNAs. Nature.

[229] Lam M.T., Li W., Rosenfeld M.G., Glass C.K. (2014). Enhancer RNAs and regulated transcriptional programs. Trends Biochem. Sci..

[230] Jonas K., Calin G.A., Pichler M. (2020). RNA-Binding Proteins as Important Regulators of Long Non-Coding RNAs in Cancer. Int. J. Mol. Sci..

[231] Ito K.K., Watanabe K., Kitagawa D. (2020). The Emerging Role of ncRNAs and RNA-Binding Proteins in Mitotic Apparatus Formation. Noncoding RNA.

[232] Cabili M.N., Dunagin M.C., McClanahan P.D., Biaesch A., Padovan-Merhar O., Regev A., Rinn J.L., Raj A. (2015). Localization and abundance analysis of human lncRNAs at single-cell and single-molecule resolution. Genome Biol..

[233] Hombach S., Kretz M. (2016). Non-coding RNAs: Classification, Biology and Functioning. Adv. Exp. Med. Biol..

[234] Naganuma T., Hirose T. (2013). Paraspeckle formation during the biogenesis of long non-coding RNAs. RNA Biol..

[235] Quinodoz S., Guttman M. (2014). Long noncoding RNAs: an emerging link between gene regulation and nuclear organization. Trends Cell Biol..

[236] Tay Y., Rinn J., Pandolfi P.P. (2014). The multilayered complexity of ceRNA crosstalk and competition. Nature.

[237] Guo L.-L., Song C.-H., Wang P., Dai L.-P., Zhang J.-Y., Wang K.-J. (2015). Competing endogenous RNA networks and gastric cancer. World J. Gastroenterol..

[238] Denzler R., Agarwal V., Stefano J., Bartel D.P., Stoffel M. (2014). Assessing the ceRNA hypothesis with quantitative measurements of miRNA and target abundance. Mol. Cell.

[239] Yang A., Shao T.-J., Bofill-De Ros X., Lian C., Villanueva P., Dai L., Gu S. (2020). AGO-bound mature miRNAs are oligouridylated by TUTs and subsequently degraded by DIS3L2. Nat. Commun..

[240] Sheu-Gruttadauria J., Pawlica P., Klum S.M., Wang S., Yario T.A., Schirle Oakdale N.T., Steitz J.A., MacRae I.J. (2019). Structural Basis for Target-Directed MicroRNA Degradation. Mol. Cell.

[241] Fuchs Wightman F., Giono L.E., Fededa J.P., de la Mata M. (2018). Target RNAs Strike Back on MicroRNAs. Front. Genet..

[242] de la Mata M., Gaidatzis D., Vitanescu M., Stadler M.B., Wentzel C., Scheiffele P., Filipowicz W., Großhans H. (2015). Potent degradation of neuronal miRNAs induced by highly complementary targets. EMBO Rep..

[243] Kleaveland B., Shi C.Y., Stefano J., Bartel D.P. (2018). A Network of Noncoding Regulatory RNAs Acts in the Mammalian Brain. Cell.

[244] Bonasio R., Shiekhattar R. (2014). Regulation of transcription by long noncoding RNAs. Annu. Rev. Genet..

[245] Xu Y., Wu W., Han Q., Wang Y., Li C., Zhang P., Xu H. (2019). New Insights into the Interplay between Non-Coding RNAs and RNA-Binding Protein HnRNPK in Regulating Cellular Functions Cells 8, 62.

[246] Ferrè F., Colantoni A., Helmer-Citterich M. (2016). Revealing protein-lncRNA interaction. Brief. Bioinform..

[247] Yoon J.H., Abdelmohsen K., Kim J., Yang X., Martindale J.L., Tominaga-Yamanaka K., White E.J., Orjalo A.V., Rinn J.L., Kreft S.G. (2013). Scaffold function of long non-coding RNA HOTAIR in protein ubiquitination. Nat. Commun..

[248] Chang L., Yuan Y., Li C., Guo T., Qi H., Xiao Y., Dong X., Liu Z., Liu Q. (2016). Upregulation of SNHG6 regulates ZEB1 expression by competitively binding miR-101-3p and interacting with UPF1 in hepatocellular carcinoma. Cancer Lett..

[249] Wang X., Lai Q., He J., Li Q., Ding J., Lan Z., Gu C., Yan Q., Fang Y., Zhao X., Liu S. (2019). LncRNA SNHG6 promotes proliferation, invasion and migration in colorectal cancer cells by activating TGF-β/Smad signaling pathway via targeting UPF1 and inducing EMT via regulation of ZEB1. Int. J. Med. Sci..

[250] Zhu Y., Xing Y., Chi F., Sun W., Zhang Z., Piao D. (2018). Long noncoding RNA SNHG6 promotes the progression of colorectal cancer through sponging miR-760 and activation of FOXC1. OncoTargets Ther..

[251] Liang R., Xiao G., Wang M., Li X., Li Y., Hui Z., Sun X., Qin S., Zhang B., Du N. (2018). SNHG6 functions as a competing endogenous RNA to regulate E2F7 expression by sponging miR-26a-5p in lung adenocarcinoma. Biomed. Pharmacother..

[252] Lv P., Qiu X., Gu Y., Yang X., Xu X., Yang Y. (2019). Long non-coding RNA SNHG6 enhances cell proliferation, migration and invasion by regulating miR-26a-5p/MAPK6 in breast cancer. Biomed. Pharmacother..

[253] Wang H., Wang L., Tang L., Luo J., Ji H., Zhang W., Zhou J., Li Q., Miao L. (2020). Long noncoding RNA SNHG6 promotes proliferation and angiogenesis of cholangiocarcinoma cells through sponging miR-101-3p and activation of E2F8. J. Cancer.

[254] Sipes J.M., Murphy-Ullrich J.E., Roberts D.D. (2018). Thrombospondins: Purification of human platelet thrombospondin-1. Methods Cell Biol.

[255] Kumar M.M., Goyal R. (2017). LncRNA as a therapeutic target for angiogenesis. Curr. Top. Med. Chem..

[256] Zaslavsky A., Baek K.-H., Lynch R.C., Short S., Grillo J., Folkman J., Italiano J.E., Ryeom S. (2010). Platelet-derived thrombospondin-1 is a critical negative regulator and potential biomarker of angiogenesis. Blood.

[257] Schadler K.L., Crosby E.J., Zhou A.Y., Bhang D.H., Braunstein L., Baek K.H., Crawford D., Crawford A., Angelosanto J., Wherry E.J., Ryeom S. (2014). Immunosurveillance by antiangiogenesis: tumor growth arrest by T cell-derived thrombospondin-1. Cancer Res..

[258] Wang W., Chen G., Wang B., Yuan Z., Liu G., Niu B., Chen Y., Zhou S., He J., Xue H. (2019). Long non-coding RNA BZRAP1-AS1 silencing suppresses tumor angiogenesis in hepatocellular carcinoma by mediating THBS1 methylation. J. Transl. Med..

[259] Guo X., Yang Z., Zhi Q., Wang D., Guo L., Li G., Miao R., Shi Y., Kuang Y. (2016). Long noncoding RNA OR3A4 promotes metastasis and tumorigenicity in gastric cancer. Oncotarget.

[260] Liu G., Hu X., Zhou G. (2017). Long non-coding RNA OR3A4 promotes proliferation and migration in breast cancer. Biomed. Pharmacother..

[261] Li W., Fu Q., Man W., Guo H., Yang P. (2019). LncRNA OR3A4 participates in the angiogenesis of hepatocellular carcinoma through modulating AGGF1/akt/mTOR pathway. Eur. J. Pharmacol..

[262] Seiz L., Kotzsch M., Grebenchtchikov N.I., Geurts-Moespot A.J., Fuessel S., Goettig P., Gkazepis A., Wirth M.P., Schmitt M., Lossnitzer A. (2010). Polyclonal antibodies against kallikrein-related peptidase 4 (KLK4): immunohistochemical assessment of KLK4 expression in healthy tissues and prostate cancer. Biol. Chem..

[263] Dong Y., Stephens C., Walpole C., Swedberg J.E., Boyle G.M., Parsons P.G., McGuckin M.A., Harris J.M., Clements J.A. (2013). Paclitaxel resistance and multicellular spheroid formation are induced by kallikrein-related peptidase 4 in serous ovarian cancer cells in an ascites mimicking microenvironment. PLoS ONE.

[264] Wilkinson R., Woods K., D’Rozario R., Prue R., Vari F., Hardy M.Y., Dong Y., Clements J.A., Hart D.N.J., Radford K.J. (2012). Human kallikrein 4 signal peptide induces cytotoxic T cell responses in healthy donors and prostate cancer patients. Cancer Immunol. Immunother..

[265] Cui Z., Cui Y., Yang S., Luo G., Wang Y., Lou Y., Sun X. (2017). KLK4 silencing inhibits the growth of oral squamous cell carcinoma through Wnt/β-catenin signaling pathway. Cell Biol. Int..

[266] Tang L., Wen J.-B., Wen P., Li X., Gong M., Li Q. (2019). Long non-coding RNA LINC01314 represses cell migration, invasion, and angiogenesis in gastric cancer via the Wnt/β-catenin signaling pathway by down-regulating KLK4. Cancer Cell Int..

[267] Wu J., Meng X., Jia Y., Chai J., Wang J., Xue X., Dang T. (2020). Long non-coding RNA HNF1A-AS1 upregulates OTX1 to enhance angiogenesis in colon cancer via the binding of transcription factor PBX3. Exp. Cell Res..

[268] Zhang L.M., Wang P., Liu X.M., Zhang Y.J. (2017). LncRNA SUMO1P3 drives colon cancer growth, metastasis and angiogenesis. Am. J. Transl. Res..

[269] Kondo A., Nonaka A., Shimamura T., Yamamoto S., Yoshida T., Kodama T., Aburatani H., Osawa T. (2017). Long Noncoding RNA JHDM1D-AS1 Promotes Tumor Growth by Regulating Angiogenesis in Response to Nutrient Starvation. Mol. Cell. Biol..

[270] Zhao J., Du P., Cui P., Qin Y., Hu C., Wu J., Zhou Z., Zhang W., Qin L., Huang G. (2018). LncRNA PVT1 promotes angiogenesis via activating the STAT3/VEGFA axis in gastric cancer. Oncogene.

[271] Gao J., Yin X., Yu X., Dai C., Zhou F. (2019). Long noncoding RNA LINC00488 functions as a ceRNA to regulate hepatocellular carcinoma cell growth and angiogenesis through miR-330-5. Dig. Liver Dis.

[272] Lin J., Cao S., Wang Y., Hu Y., Liu H., Li J., Chen J., Li P., Liu J., Wang Q., Zheng L. (2018). Long non-coding RNA UBE2CP3 enhances HCC cell secretion of VEGFA and promotes angiogenesis by activating ERK1/2/HIF-1α/VEGFA signalling in hepatocellular carcinoma. J. Exp. Clin. Cancer Res..

[273] Yuan S.X., Yang F., Yang Y., Tao Q.F., Zhang J., Huang G., Yang Y., Wang R.Y., Yang S., Huo X.S. (2012). Long noncoding RNA associated with microvascular invasion in hepatocellular carcinoma promotes angiogenesis and serves as a predictor for hepatocellular carcinoma patients’ poor recurrence-free survival after hepatectomy. Hepatology.

[274] Hou Z.H., Xu X.W., Fu X.Y., Zhou L.D., Liu S.P., Tan D.M. (2020). Long non-coding RNA MALAT1 promotes angiogenesis and immunosuppressive properties of HCC cells by sponging miR-140. Am. J. Physiol. Cell Physiol..

[275] Jeck W.R., Sorrentino J.A., Wang K., Slevin M.K., Burd C.E., Liu J., Marzluff W.F., Sharpless N.E. (2013). Circular RNAs are abundant, conserved, and associated with ALU repeats. RNA.

[276] Zhang Y., Zhang X.-O., Chen T., Xiang J.-F., Yin Q.-F., Xing Y.-H., Zhu S., Yang L., Chen L.L. (2013). Circular intronic long noncoding RNAs. Mol. Cell.

[277] Zhang X.-O., Wang H.-B., Zhang Y., Lu X., Chen L.-L., Yang L. (2014). Complementary sequence-mediated exon circularization. Cell.

[278] Zhang Y., Xue W., Li X., Zhang J., Chen S., Zhang J.L., Yang L., Chen L.L. (2016). The biogenesis of nascent circular RNAs. Cell Rep..

[279] Liang D., Wilusz J.E. (2014). Short intronic repeat sequences facilitate circular RNA production. Genes Dev..

[280] Conn S.J., Pillman K.A., Toubia J., Conn V.M., Salmanidis M., Phillips C.A., Roslan S., Schreiber A.W., Gregory P.A., Goodall G.J. (2015). The RNA binding protein quaking regulates formation of circRNAs. Cell.

[281] Aktaş T., Avşar Ilık İ., Maticzka D., Bhardwaj V., Pessoa Rodrigues C., Mittler G., Manke T., Backofen R., Akhtar A. (2017). DHX9 suppresses RNA processing defects originating from the Alu invasion of the human genome. Nature.

[282] Ashwal-Fluss R., Meyer M., Pamudurti N.R., Ivanov A., Bartok O., Hanan M., Evantal N., Memczak S., Rajewsky N., Kadener S. (2014). circRNA biogenesis competes with pre-mRNA splicing. Mol. Cell.

[283] Tang C., Xie Y., Yu T., Liu N., Wang Z., Woolsey R.J., Tang Y., Zhang X., Qin W., Zhang Y. (2020). m^6^A-dependent biogenesis of circular RNAs in male germ cells. Cell Res..

[284] Panda A.C., Grammatikakis I., Munk R., Gorospe M., Abdelmohsen K. (2017). Emerging roles and context of circular RNAs. Wiley Interdiscip. Rev. RNA.

[285] Zhang Q., Jin X.S., Yang Z.Y., Wei M., Liu B.Y., Gu Q.L. (2013). Upregulated Hoxc6 expression is associated with poor survival in gastric cancer patients. Neoplasma.

[286] Zhou J., Yang X., Song P., Wang H., Wang X. (2019). *HOXC6* in the prognosis of prostate cancer. Artif. Cells Nanomed. Biotechnol..

[287] Shi H., Li H., Zhen T., Dong Y., Pei X., Zhang X. (2020). hsa_circ_001653 Implicates in the Development of Pancreatic Ductal Adenocarcinoma by Regulating MicroRNA-377-Mediated HOXC6 Axis. Mol. Ther. Nucleic Acids.

[288] Varisli L., Gonen-Korkmaz C., Debelec-Butuner B., Erbaykent-Tepedelen B., Muhammed H.S., Bogurcu N., Saatcioglu F., Korkmaz K.S. (2011). Ubiquitously expressed hematological and neurological expressed 1 downregulates Akt-mediated GSK3β signaling, and its knockdown results in deregulated G2/M transition in prostate cells. DNA Cell Biol..

[289] Li L., Zeng T.-T., Zhang B.-Z., Li Y., Zhu Y.-H., Guan X.-Y. (2017). Overexpression of HN1L promotes cell malignant proliferation in non-small cell lung cancer. Cancer Biol. Ther..

[290] Chen J.J., Sun X., Mao Q.Q., Jiang X.Y., Zhao X.G., Xu W.J., Zhong L. (2020). Increased expression of hematological and neurological expressed 1 (HN1) is associated with a poor prognosis of hepatocellular carcinoma and its knockdown inhibits cell growth and migration partly by down-regulation of c-Met. Kaohsiung J. Med. Sci..

[291] Pu J., Wang J., Li W., Lu Y., Wu X., Long X., Luo C., Wei H. (2020). hsa_circ_0000092 promotes hepatocellular carcinoma progression through up-regulating HN1 expression by binding to microRNA-338-3p. J. Cell. Mol. Med.

[292] Huang X.Y., Huang Z.L., Huang J., Xu B., Huang X.Y., Xu Y.H., Zhou J., Tang Z.Y. (2020). Exosomal circRNA-100338 promotes hepatocellular carcinoma metastasis via enhancing invasiveness and angiogenesis. J. Exp. Clin. Cancer Res..

[293] Yu Y.X., Ge T.W., Zhang P. (2020). Circular RNA circGFRA1 promotes angiogenesis, cell proliferation and migration of hepatocellular carcinoma by combining with miR-149. Eur. Rev. Med. Pharmacol. Sci..

[294] Girardi E., López P., Pfeffer S. (2018). On the Importance of Host MicroRNAs During Viral Infection. Front. Genet..

[295] Chen G., Wang Z., Wang D., Qiu C., Liu M., Chen X., Zhang Q., Yan G., Cui Q. (2013). LncRNADisease: a database for long-non-coding RNA-associated diseases. Nucleic Acids Res..

[296] Wang J., Zhang X., Chen W., Li J., Liu C. (2018). CRlncRNA: a manually curated database of cancer-related long non-coding RNAs with experimental proof of functions on clinicopathological and molecular features. BMC Med. Genomics.

[297] Gao Y., Wang P., Wang Y., Ma X., Zhi H., Zhou D., Li X., Fang Y., Shen W., Xu Y. (2019). Lnc2Cancer v2.0: updated database of experimentally supported long non-coding RNAs in human cancers. Nucleic Acids Res..

[298] Hu G., Drescher K.M., Chen X.M. (2012). Exosomal miRNAs: Biological Properties and Therapeutic Potential. Front. Genet..

[299] Wei L., Sun J., Zhang N., Zheng Y., Wang X., Lv L., Liu J., Xu Y., Shen Y., Yang M. (2020). Noncoding RNAs in gastric cancer: implications for drug resistance. Mol. Cancer.

[300] Pourhanifeh M.H., Mahjoubin-Tehran M., Shafiee A., Hajighadimi S., Moradizarmehri S., Mirzaei H., Asemi Z. (2020). MicroRNAs and exosomes: Small molecules with big actions in multiple myeloma pathogenesis. IUBMB Life.

[301] Liu Y., Wang J. (2016). Therapeutic Potentials of Noncoding RNAs: Targeted Delivery of ncRNAs in Cancer Cells. Adv. Exp. Med. Biol..

[302] Segal M., Slack F.J. (2020). Challenges identifying efficacious miRNA therapeutics for cancer. Expert Opin. Drug Discov..

[303] Ning B., Yu D., Yu A.-M. (2019). Advances and challenges in studying noncoding RNA regulation of drug metabolism and development of RNA therapeutics. Biochem. Pharmacol..

